# Supplementary material for: A CYBDOM protein impacts iron homeostasis and primary root growth under phosphate deficiency in Arabidopsis
Source: Nat Commun. 2024 Jan 11;15:423. doi: 10.1038/s41467-023-43911-x (PMC10784552; doi:10.1038/s41467-023-43911-x)
Supplement: Supplementary file 1 — Supplementary Information [file 41467_2023_43911_MOESM1_ESM.pdf]

Supplementary Figures

A CYBDOM protein impacts iron homeostasis and primary root growth under phosphate deficiency in Arabidopsis

Joaquín Clúa, Jonatan Montpetit, Pedro Jimenez-Sandoval, Christin Naumann, Julia Santiago, and Yves Poirier

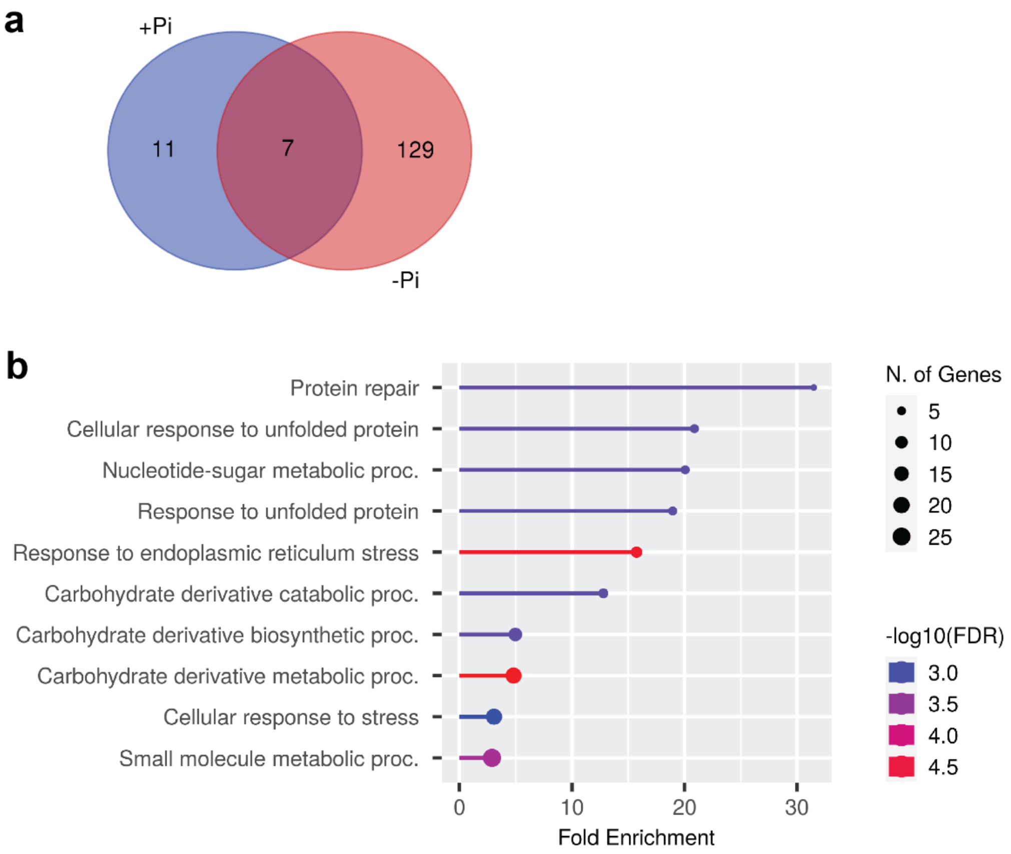

c

AT3G45600	Member of TETRASPANIN family (TET3)
AT3G25290	Cytochrome and domon domain containing protein
AT1G76090	Sterol methyltransferase 3 (SMT3)
AT2G28190	Copper zinc superoxide dismutase 2 (CSD2)
AT4G16660	Heat shock protein 70 (Hsp 70) family protein
AT5G13640	Phospholipid:diacylglycerol acyltransferase (PDAT)
AT1G04910	O-fucosyltransferase family protein

Supplementary Fig. 1 Proteomic analysis of *cnx1 cnx2* double mutant.
a, Venn diagram showing the differentially abundant proteins between Col-0 and *cnx1 cnx2* in plus (+Pi) and minus (-Pi) phosphate conditions. Plants were grown for 7 days in +Pi and -Pi and root tissue was processed for a proteomic analysis based on iTRAQ labeling and LC-MS/MS. For each condition differential abundant proteins between Col-0 and *cnx1 cnx2* were identified according to the following criteria: p-value ≤0.05 Student's T-test; -1.5≥ FC ≥1.5. **b**, Gene ontology (GO) enrichment analysis of the differentially abundant proteins between Col-0 and *cnx1 cnx2*. The top 10 enriched 'molecular function' GO terms are shown in a lollipop graph where the fold enrichment, number of the corresponding genes (N. of genes) and the fold discovery rate (FDR) are indicated. **c**, Table showing the seven proteins that were differentially abundant between Col-0 and *cnx1 cnx2* both in +P and -Pi. The gene identification number and the annotation are shown.

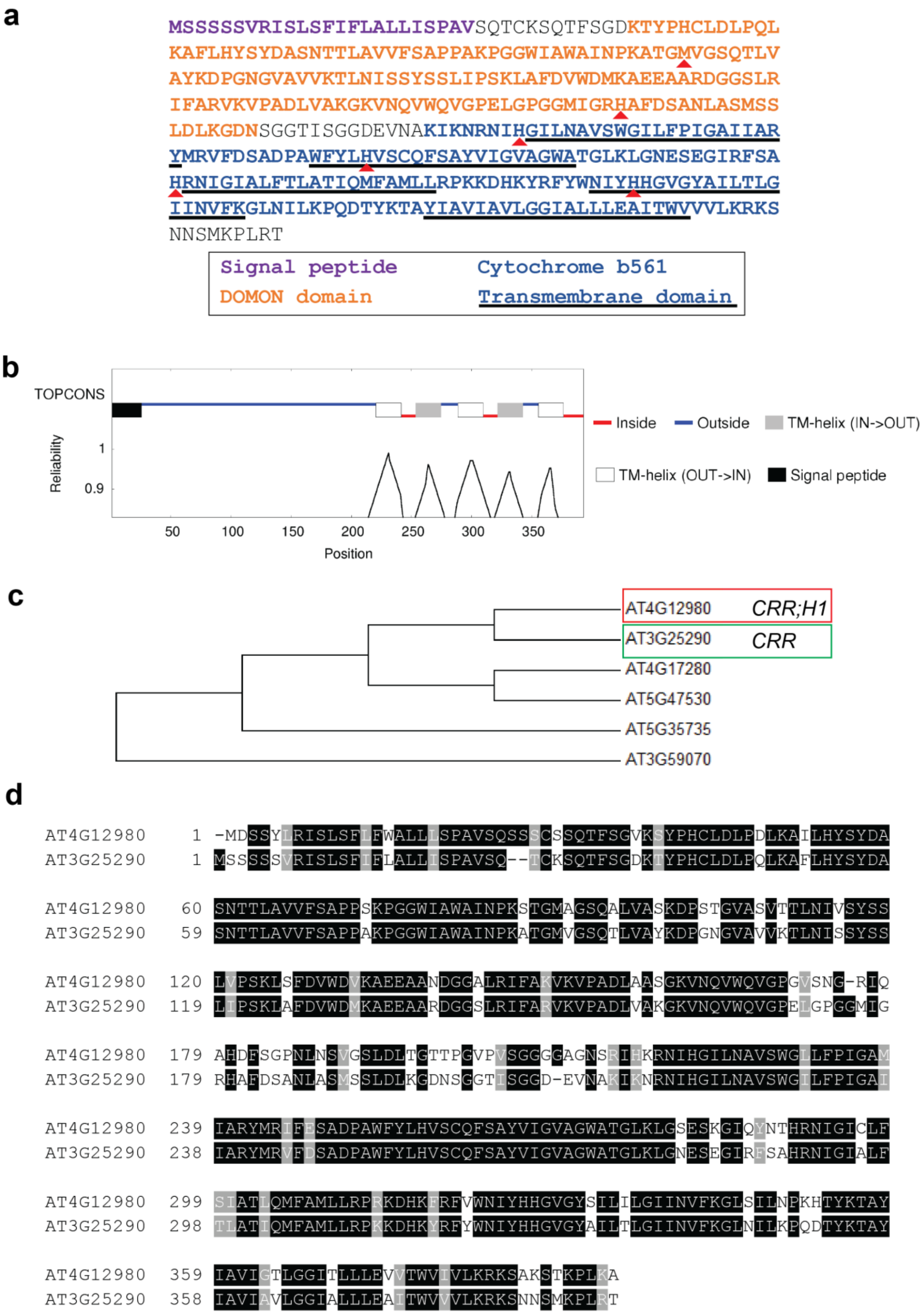
a
MSSSSSVRISLSFIFLALLISPAVSQTCKSQTFSGDKTYPHCLDLPQL
KAFLHYSYDASNTTLAVVFSAPPAKPGGWIAWAINPKATGMVGSQTLV
AYKDPGNGVAVVKTLNISSYSSLIPSKLAFDVWDMKAEEAARDGGSLR
IFARVKVPADLVAKGKVNQVWQVGPELGPGGMIGRHAFDSANLASMSS
LDLKGDNSGGTISGGDEVNAKIKNRNIHGILNAVSWGILFPIGAIIAR
YMRVFDSADPAWFYLHVSCQFSAYVIGVAGWATGLKLGNESEGIRFSA
HRNIGIALFTLATIQMFAMLLRPKKDHKYRFYWNIYHHGVGYAILTLG
IINVFKGLNILKPQDTYKTAYIAVIAVLGGIALLLEAITWVVVLKRKS
NNSMKPLRT
Signal peptide
Cytochrome b561
DOMON domain
Transmembrane domain
b
TOPCONS
Reliability
1
0.9
50
100
150
200
250
300
350
Position
Inside
Outside
TM-helix (IN->OUT)
TM-helix (OUT->IN)
Signal peptide
c
AT4G12980
CRR;H1
AT3G25290
CRR
AT4G17280
AT5G47530
AT5G35735
AT3G59070
d
AT4G12980 1 -MDSSYLRISLSFLFWALLLSPAVSQSSSCSSQTFSGVKSYPHCLDLPDLKAILHYSYDA
AT3G25290 1 MSSSSSVRISLSFIFLALLISPAVSQ--TCKSQTFSGDKTYPHCLDLPQLKAFLHYSYDA
AT4G12980 60 SNTTLAVVFSAPPSKPGGWIAWAINPKSTGMAGSQALVASKDPSTGVASVTTLNIVSYSS
AT3G25290 59 SNTTLAVVFSAPPAKPGGWIAWAINPKATGMVGSQTLVAYKDPGNGVAVVKTLNISSYSS
AT4G12980 120 LVPSKLSFDVWDVKAEEAANDGGALRIFAKVKVPADLAASGKVNQVWQVGPGVSNG-RIQ
AT3G25290 119 LIPSKLAFDVWDMKAEEAARDGGSLRIFARVKVPADLVAKGKVNQVWQVGPELGPGGMIG
AT4G12980 179 AHDFSGPNLNSVGSLDLTGTTPGVPVSGGGAGNSRIHKRNIHGILNAVSWGLLFPIGAM
AT3G25290 179 RHAFDSANLASMSSLDLKGDNSGGTISGGD-EVNAKIKNRNIHGILNAVSWGILFPIGAI
AT4G12980 239 IARYMRIFESADPAWFYLHVSCQFSAYVIGVAGWATGLKLGSESKGIQYNTHRNIGICLF
AT3G25290 238 IARYMRVFDSADPAWFYLHVSCQFSAYVIGVAGWATGLKLGNESEGIRFSAHRNIGIALF
AT4G12980 299 SIATLQMFAMLLRPRKDHKFRFVWNIYHHGVGYSILILGIINVFKGLSILNPKHTYKTAY
AT3G25290 298 TLATIQMFAMLLRPKKDHKYRFYWNIYHHGVGYAILTLGIINVFKGLNILKPQDTYKTAY
AT4G12980 359 IAVIGTLGGITLLLEVVTWVIVLKRKSAKSTKPLKA
AT3G25290 358 IAVIAVLGGIALLLEAITWVVVLKRKSNNSMKPLRT

Supplementary Fig. 2 CRR protein features and phylogeny.

a, CRR amino acid sequence showing the location of the signal peptide (SP), the DOMON, cytochrome b561 and transmembrane (TM) domains. The conserved methionine (M) and histidine (H) of the DOMON domain as well as the four conserved histidines of the cytochrome b561 are indicated by red triangles. **b**, CRR topology and transmembrane domains as predicted by the TOPCONS software (topcons.cbr.su.se). **c**, Phylogenetic tree showing the 6 Arabidopsis CYBDOM members belonging to the F2 clade. CRR and CRR;H1 are highlighted in green and red, respectively. The tree was constructed using the neighbor-joining method based on the multiple sequence alignment analysis of the full-length proteins. **d**, Protein sequence alignment of CRR and CRR;H1. The alignment was generated using ClustalOmega and the conserved regions highlighted using BOXSHADE.

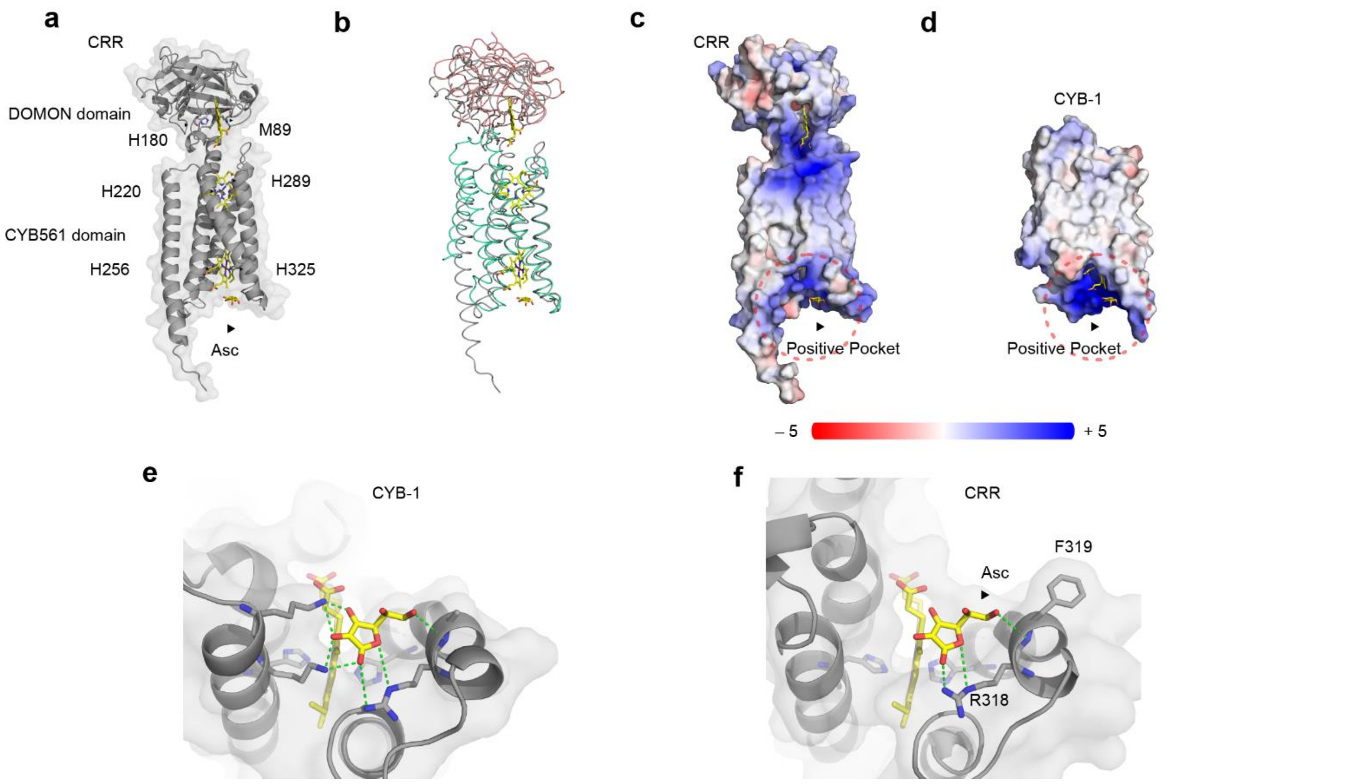

Supplementary Fig. 3 Structural analysis of the CRR protein model.

a, Cartoon representation of the CRR structural model with superimposed b heme groups and ascorbate from the cellobiose dehydrogenase from *Phanerodontia chrysosporium* (PDB ID 1D7B) and the CYB561 domain structure of the ascorbate-dependent oxidoreductase CYB-1 from *Arabidopsis thaliana* (PDB ID 4O79). The CRR DOMON domain contains the conserved residues Met89 and His180 to coordinate the superimposed b heme. The CYB561 domain accommodates two b hemes between the transmembrane alpha helixes, coordinated by the conserved pairs His220 - His289, and His256 - His325, respectively. **b**, Superimposition of the CRR structural model (in grey) with the crystal structures of the DOMON domain from the cellobiose dehydrogenase from *Phanerodontia chrysosporium* (PDB ID 1D7B, shown in pink) (RMSD = 1.055 Å, comparing 179 pairs of Cα atoms), and the CYB561 domain of the ascorbate-dependent oxidoreductase CYB-1 from Arabidopsis thaliana (PDB ID 4O79, shown in cyan) (RMSD = 1.247 Å, comparing 163 pairs of Cα atoms). **c** and **d**, Electrostatic potential surface analysis reveals a putative ascorbate binding pocket in the CRR protein. The CRR protein contains a conserved positively charged patch located in the cytosolic region, that binds ascorbate in the CYB-1 enzyme (PDB ID 4O79). The patch is indicated with a red dotted circle. **e** and **f**, The basic cytoplasmic patch in the CYB561 domain of the CRR protein folds into a similar architecture and contains conserved residues required to coordinate ascorbate in the CYB-1 protein. The coordinating residues are depicted in sticks and the interactions indicated with green dotted lines. **e**, Structural details of the ascorbate binding pocket in the CYB-1 protein (PDB ID 4O79). The ascorbate molecule is coordinated via side chain and backbone polar interactions. **f**, Structural details of the positively charged patch in the CRR CYB561 domain with superimposed ascorbate from the CYB-1 oxidoreductase (PDB ID 4O79). The main arginine residue (Arg318) and backbone interactions responsible for ascorbic acid binding are conserved.

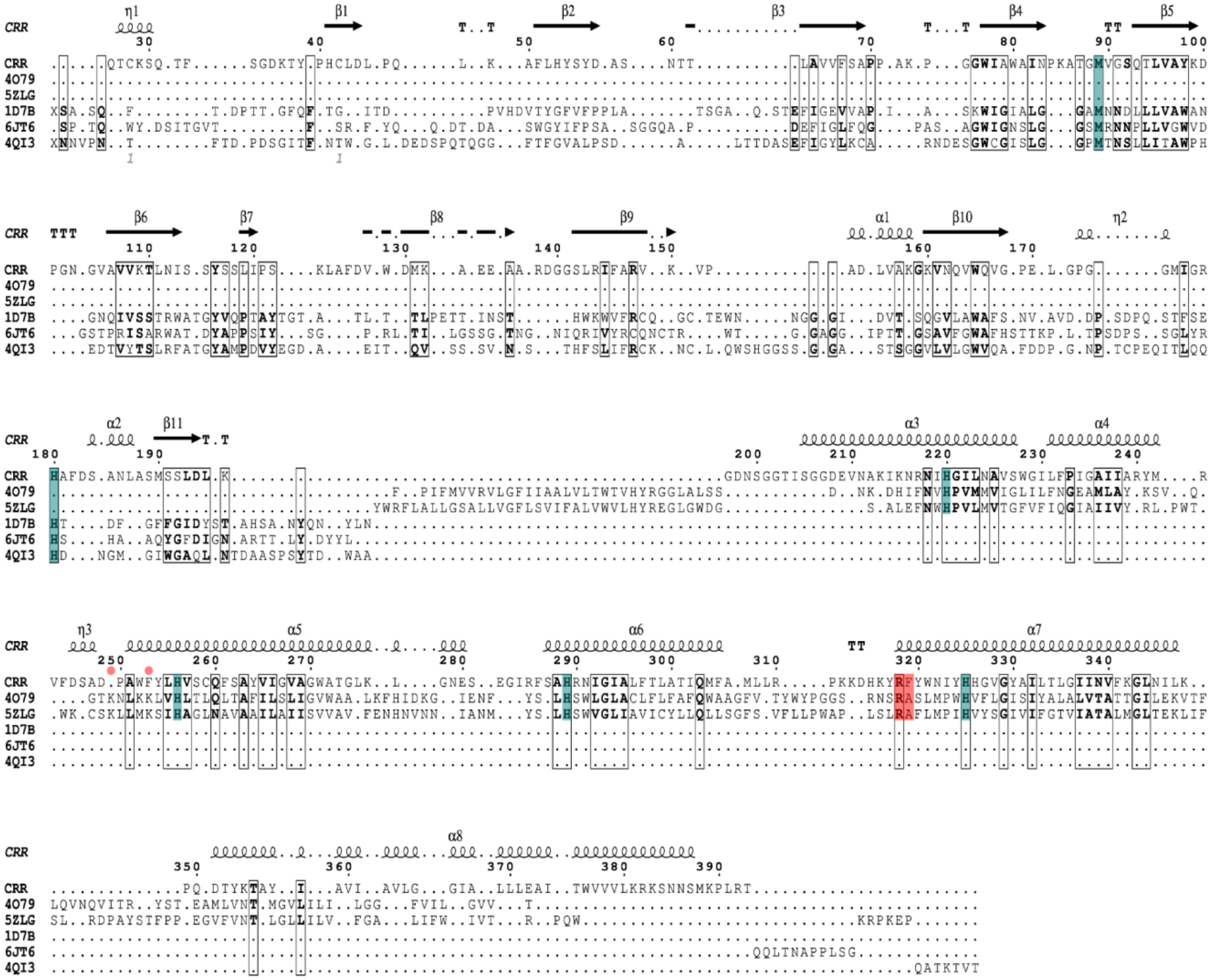

Supplementary Fig. 4 Structural signatures required for b heme and ascorbic acid binding are conserved among different DOMON and CYB561 containing proteins.

Structure-based sequence alignment of the CRR AlphaFold model and the crystal structures of DOMON domain containing proteins: cellobiose dehydrogenase of *Phanerodontia chrysosporium* (PDB ID 1D7B), pyranose dehydrogenase from *Coprinopsis cinerea* (PDB ID 6JT6), cellobiose dehydrogenase of *Myriococcum thermophilum* (PDB ID 4QI3), and CYB561 domain containing proteins: cytochrome b561 from *Arabidopsis thaliana* (PDB ID 4O79) and human duodenal cytochrome b (PDB ID 5ZLG). Conserved residues for b heme group coordination are highlighted in cyan. The conserved interactions with ascorbate are highlighted in red. The two Lys that coordinate ascorbate in the CYB-1 enzyme (PDB : 4O79) that are not conserved in the CRR protein are indicated with a red dot.

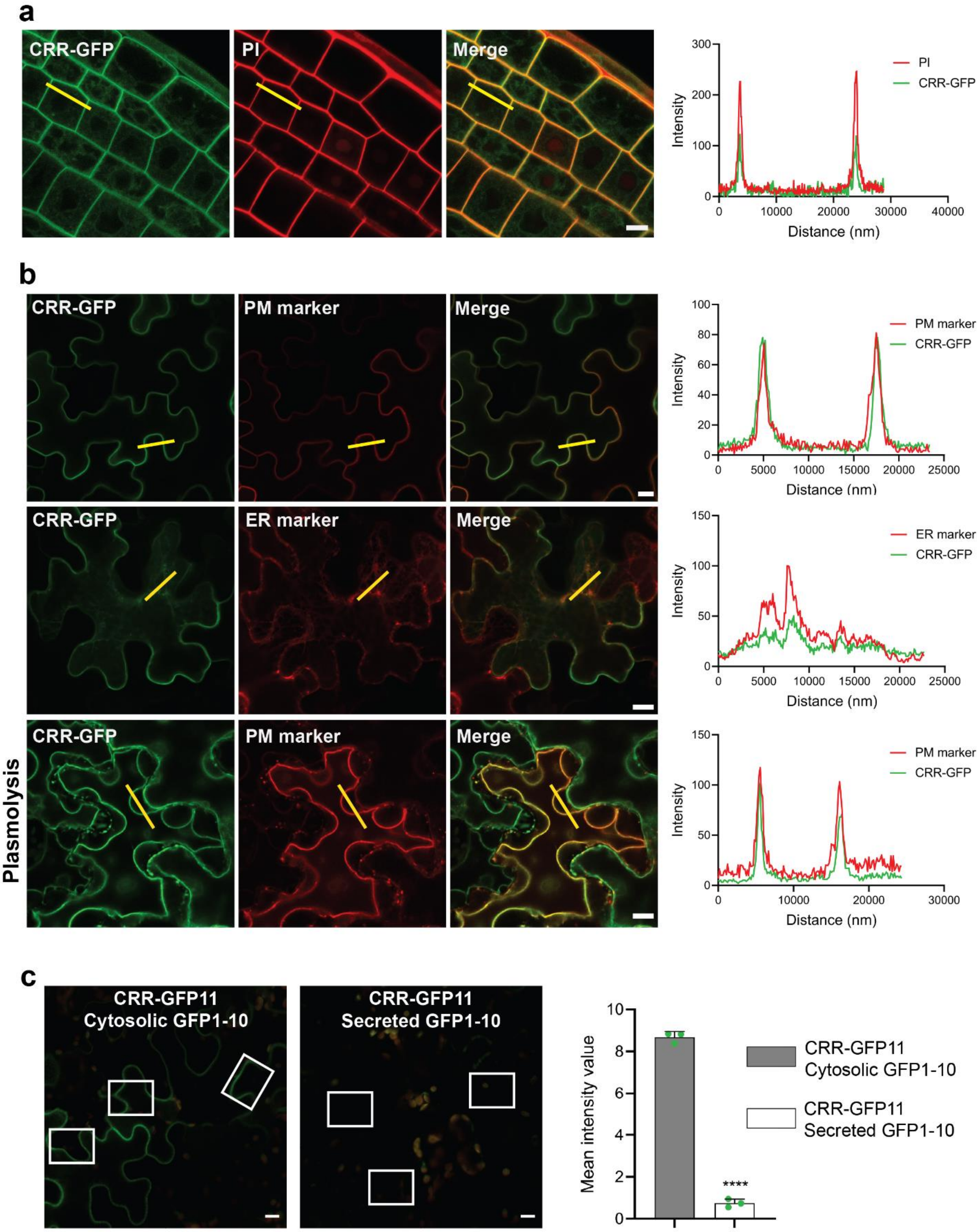

a
CRR-GFP
PI
Merge
PI
CRR-GFP
Intensity
Distance (nm)
b
CRR-GFP
PM marker
Merge
PM marker
CRR-GFP
Intensity
Distance (nm)
CRR-GFP
ER marker
Merge
ER marker
CRR-GFP
Plasmolysis
CRR-GFP
PM marker
Merge
PM marker
CRR-GFP
c
CRR-GFP11
Cytosolic GFP1-10
CRR-GFP11
Secreted GFP1-10
Mean intensity value
****
CRR-GFP11
Cytosolic GFP1-10
CRR-GFP11
Secreted GFP1-10

Supplementary Fig. 5 CRR subcellular localization and topology analysis.

a, CRR primarily localizes to the plasma membrane in *Arabidopsis thaliana*. The confocal microscopy images show an Arabidopsis transgenic root expressing a translational fusion CRR-GFP driven by the 35S promotor (green channel) together with the cell wall marker propidium iodide (PI) in red. The merged channels show the co-localization between CRR and PI. **b**, Subcellular localization of CRR in *Nicotiana benthamiana*. Confocal images showing *N. benthamiana* epidermal cells expressing CRR-GFP together with the plasma membrane marker CBL1 (top panels) fused to the orange fluorescent protein or the ER marker ER-rk fused to mCherry (middle panels). In order to confirm CRR plasma membrane localization cells were plasmolyzed by the addition of NaCl (bottom panels). In (a) and (b), GFP and PI signal intensity profile was plotted along the indicated yellow line, showing the co-localization between CRR and the subcellular markers. **c**, Split-GFP system showing that CRR C-terminus is facing the cytosol. The 11th beta-strand of the GFP was translationally fused to the C-terminus of CRR (CRR-GFP11) and co-expressed in *N. benthamiana* together with the other 10 GFP beta-strands targeted either to the cytosol (cytosolic GFP1-10) or the apoplast (secreted GFP1-10). GFP fluorescence was only detected by confocal microscopy when CRR-GFP11 was co-expressed with the cytosolic GFP1-10, indicating that the CRR C-terminus is intracellular. GFP signal was quantified as mean intensity value in the regions indicated by white boxes. Bars represent the mean ± sd (sample size is indicated by green dots; n=3). Statistical analysis was performed by an unpaired two-tailed t-test (****$P < 0.0001$). In (a), (b) and (c) scale is 10 µm. Source data, including n and p values, are provided as a Source Data file.

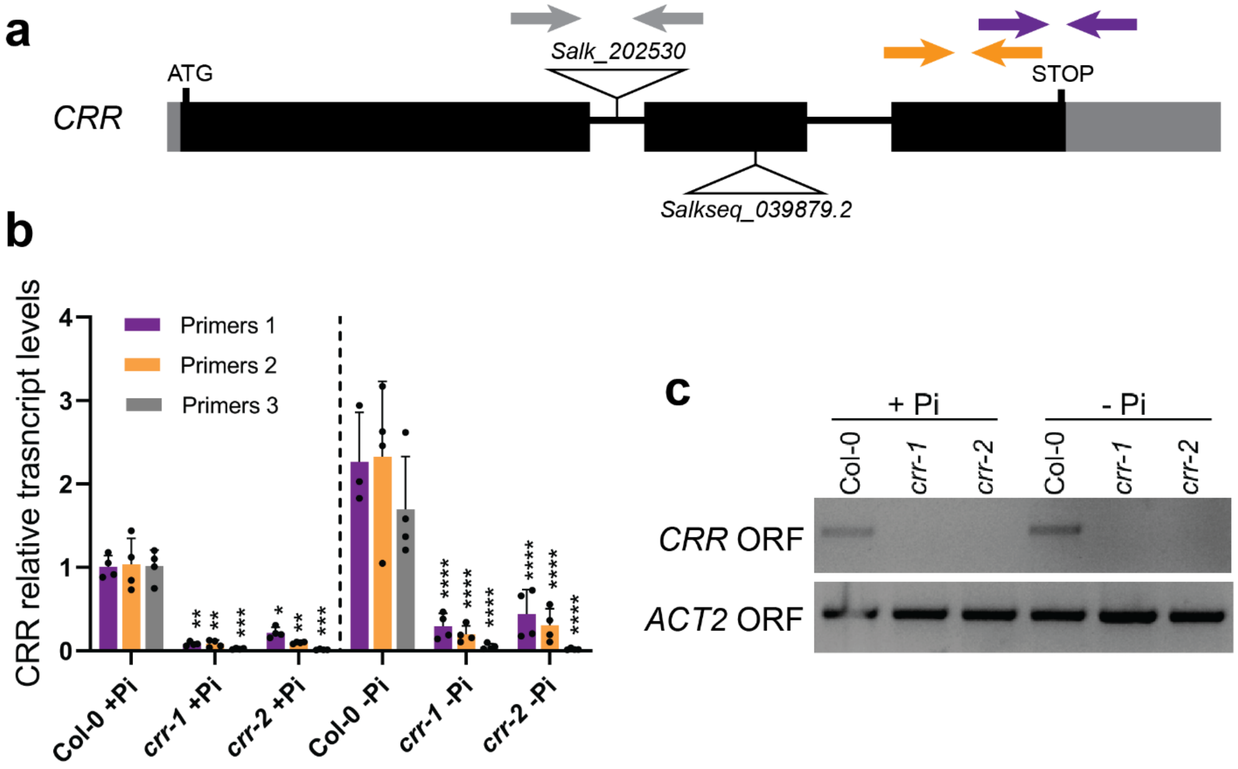

Supplementary Fig. 6 Characterization of *Arabidopsis thaliana* CRR T-DNA mutants.
a, Scheme showing the *CRR* gene structure and the localization of two different T-DNA insertions. Grey boxes indicate the 5'- and 3'-UTRs, black boxes represent the 3 exons and the black lines connecting them the introns. The *A. thaliana* line Salk_202530 (*crr-1*) has a T-DNA insertion in the first intron while the line Salkseq_039879.2 (*crr-2*) has an insertion in the second exon. The grey, orange, and purple arrows indicate the positions of the primers used in (b). **b**, The two *CRR* T-DNA mutants *crr-1* and *crr-2* have reduced levels of *CRR* mRNA. Arabidopsis Col-0, *crr-1* and *crr-2* seedlings were grown for 7 days in plus (+Pi) or minus (-Pi) phosphate conditions and *CRR* relative transcript levels were quantified by qRT-PCR (RNA was extracted from whole seedlings) using the three different set of primers shown in (a). Data are presented as means ± sd and the sample size is indicated by black dots; n=4 in all cases and 3 for Col-0 -Pi Primer 1. Statistical analysis was performed by a two-way ANOVA followed by a Tukey's test and significant differences against Col-0 under +Pi or -Pi are indicated by asterisks (****P <0.0001; ***P < 0.001; **P < 0.01; *P < 0.05). **c**, Full length *CRR* complementary DNA (cDNA) is undetectable in the *crr* T-DNA mutants. cDNA obtained from Col-0, *crr-1* and *crr-2* grown in +Pi and -Pi was utilized as template for PCR reactions using primers to amplify either *CRR* or *ACT2* complete open reading frames (ORF). The PCR products were resolved by agarose gel electrophoresis and revealed by ethidium bromide staining. While *ACT2* was detected in all samples, *CRR* ORF was only detected in Col-0 samples. Source data, including n and p values, are provided as a Source Data file.

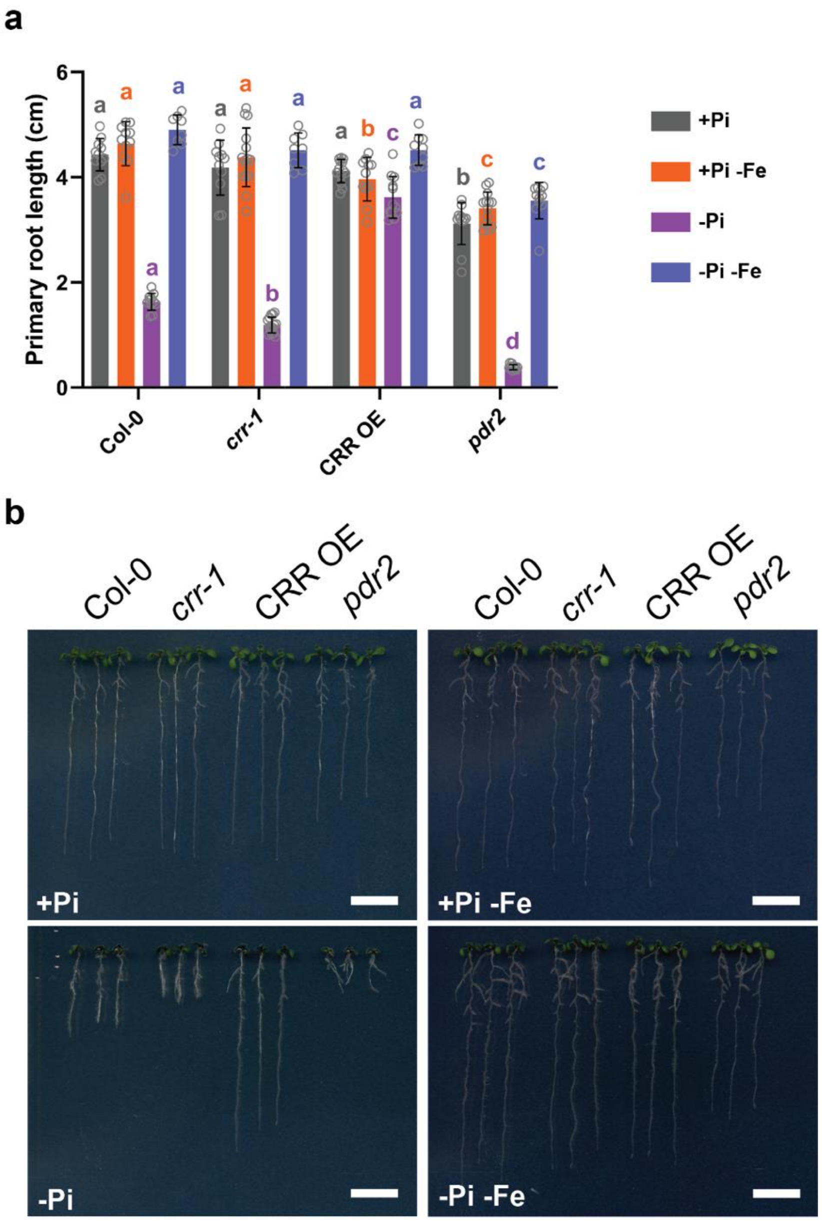

Supplementary Fig. 7 *crr* primary root growth phenotype under phosphate starvation is iron dependent.

a, Primary root length quantification of Col-0, *crr-1*, CRR OE and *pdr2* seedlings grown in plus (+Pi) or minus phosphate (-Pi) with or without the addition of ferrozine to sequester the iron in the media. Media with the addition of ferrozine is indicated as (-Fe). The data is represented as mean ± s.d. (sample size is indicated by circles) and a One-way ANOVA followed by a Tukey's multiple comparisons test was used to assess the statistical differences. Significant differences ($P < 0.05$) within each condition (same color code) are indicated by different letters. **b**, Photographs showing representative 7 days post germination seedlings grown in the conditions indicated in (a). Scale: 1cm. Source data, including n and p values, are provided as a Source Data file.

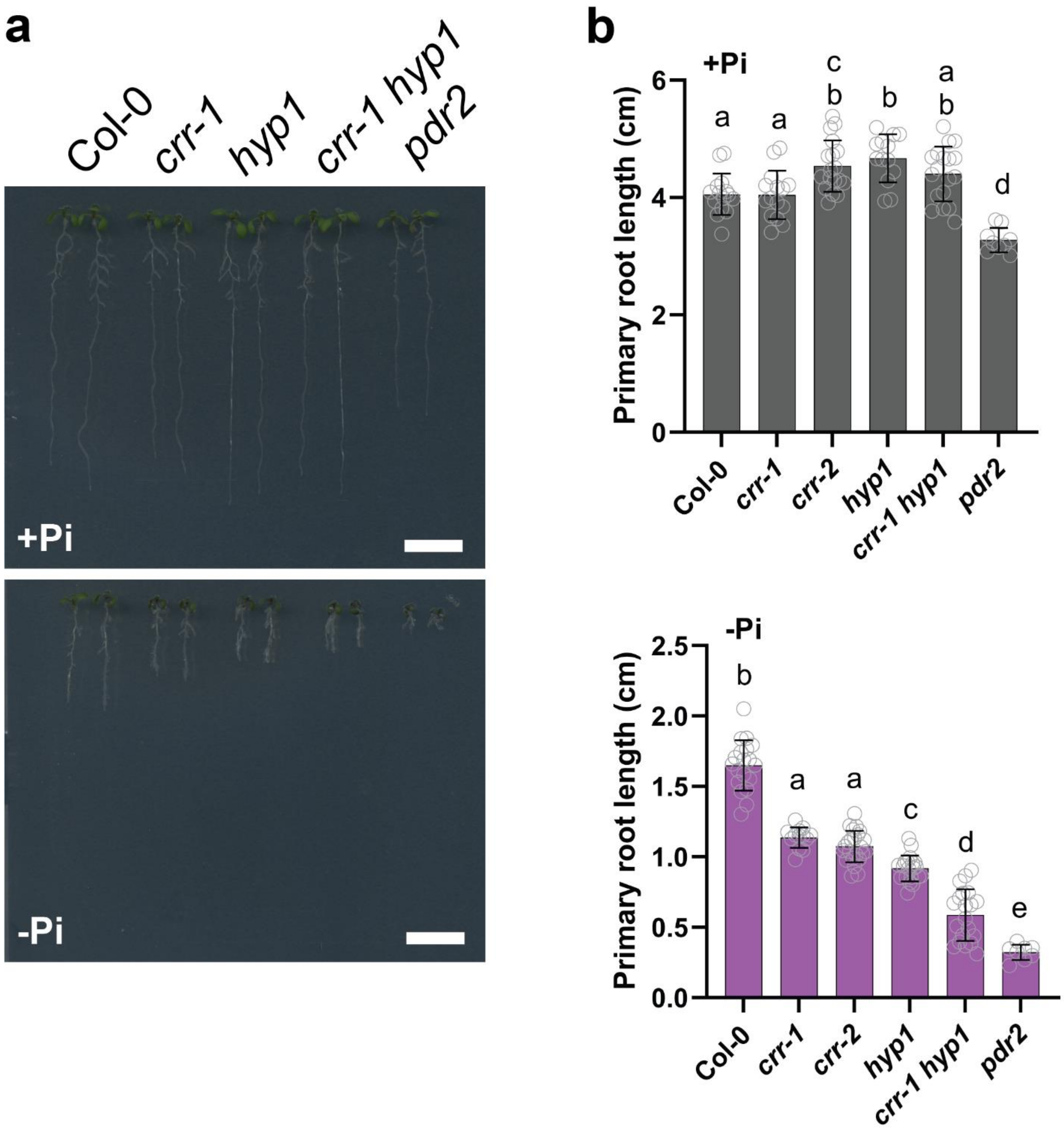

Supplementary Fig. 8 *CRR* and *HYP1* are involved in primary root growth inhibition under Pi deficiency.

a, Phenotype of Col-0, *crr-1*, *hyp1*, *crr-1 hyp1* double mutant, and *pdr2* under plus (+Pi) and minus (-Pi). Scale: 1cm. **b**, Quantification of the primary root growth shown as means ± sd (sample size is indicated by circles). Statistical analysis was performed by a One-way ANOVA followed by a Tukey's test. Different letters indicate significant differences ($P < 0.05$). Source data, including n and p values, are provided as a Source Data file.

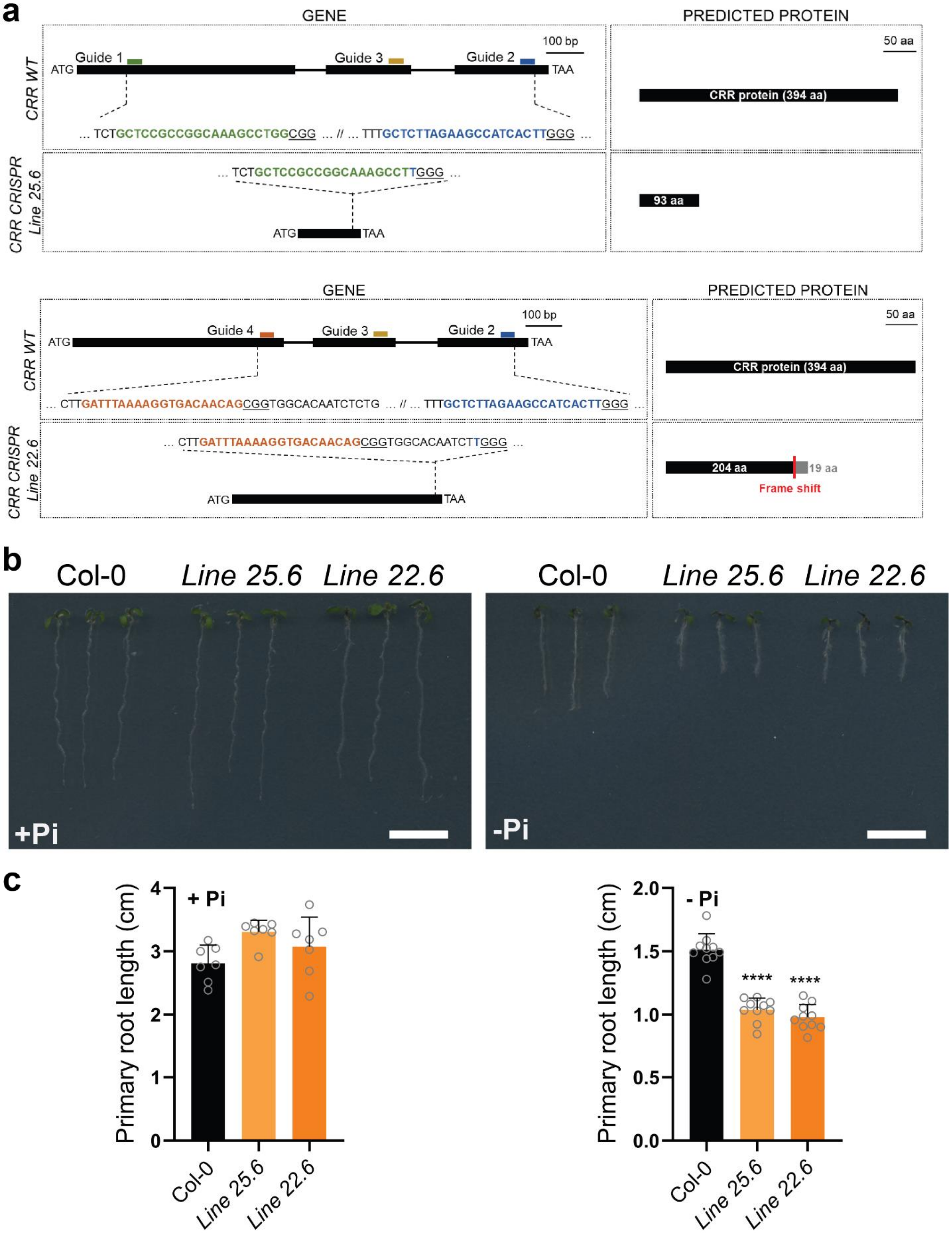
a
GENE
PREDICTED PROTEIN
CRR WT
Guide 1
Guide 3
Guide 2
100 bp
50 aa
ATG
TAA
... TCTGCTCCGCCGGCAAAGCCTGGCGG ... // ... TTTGCTCTTAGAAGCCATCACTTGGG ...
CRR protein (394 aa)
CRR CRISPR Line 25.6
... TCTGCTCCGCCGGCAAAGCCTTGGG ...
93 aa
GENE
PREDICTED PROTEIN
Guide 4
... CTTGATTTAAAAGGTGACAACAGCGGTGGCACAATCTCTG ... // ... TTTGCTCTTAGAAGCCATCACTTGGG ...
CRR CRISPR Line 22.6
... CTTGATTTAAAAGGTGACAACAGCGGTGGCACAATCTTGGG ...
204 aa
19 aa
Frame shift
b
Col-0
Line 25.6
Line 22.6
+Pi
-Pi
c
Primary root length (cm)
+ Pi
- Pi
****
Col-0
Line 25.6
Line 22.6

Supplementary Fig. 9 *crr* mutants generated by CRISPR/Cas9 show a short root phenotype.
a, Schematic representation of the strategy used to edit the *CRR* locus by CRISPR/Cas9 and the resulting edited lines. Two pools of Arabidopsis plants were transformed with different sets of guide RNAs (guides 1, 2, and 3 or guides 2, 3 and 4) targeting the *CRR* locus. The analysis of independent T1 plants allowed the identification of two independent edited lines. Line 25.6 has an in-frame deletion of 1120 bases in the *CRR* gene, generating an open reading frame coding for an hypothetical protein of 93 amino acids (top panel). Line 22.6 bears a frame-shift mutation of 729 nucleotides generating an hypothetical ORF coding for a protein of 223 amino acids where the first 204 corresponds to the DOMON domain (bottom panels). **b**, Pictures showing the root phenotype of the *crr* edited lines grown in plus (+Pi) and (-Pi) minus phosphate conditions. Scale: 1 cm. **c**, Primary root length quantification of Col-0 and the *crr* edited lines 7 days after germination. Data represents the mean± s.d. (sample size is indicated by circles; n=7 for +Pi and 10 for -Pi) and the statistical differences were analyzed using a One-way ANOVA followed by a Tukey's multiple comparisons test. Significant differences against Col-0 are indicated (***$P < 0.001$; **$P < 0.01$; *$P < 0.05$). Source data, including n and p values, are provided as a Source Data file.

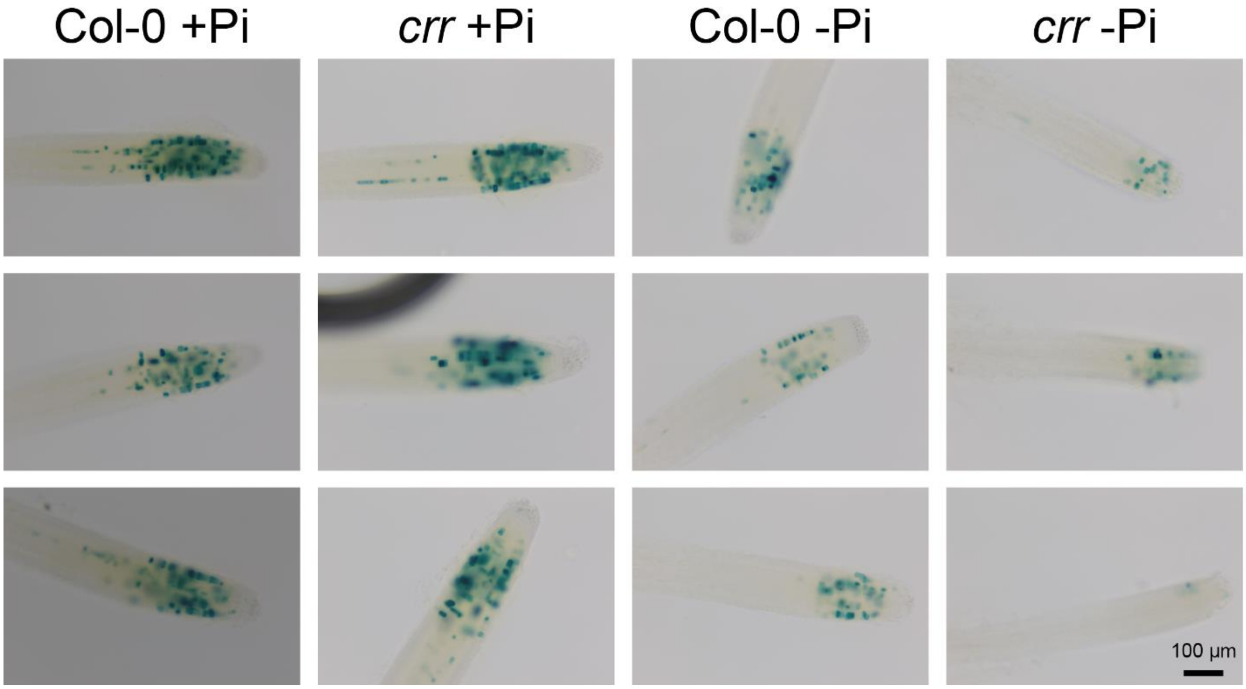

Supplementary Fig. 10 CYCB1 expression in the root apical meristem is reduced in *crr* mutant under phosphate deficiency.

Representative images showing variability of the histochemical analysis of the G2/M transition marker *pCYCB1::GUS* in Col-0 and *crr-1* root apical meristems. Scale = 100 µm.

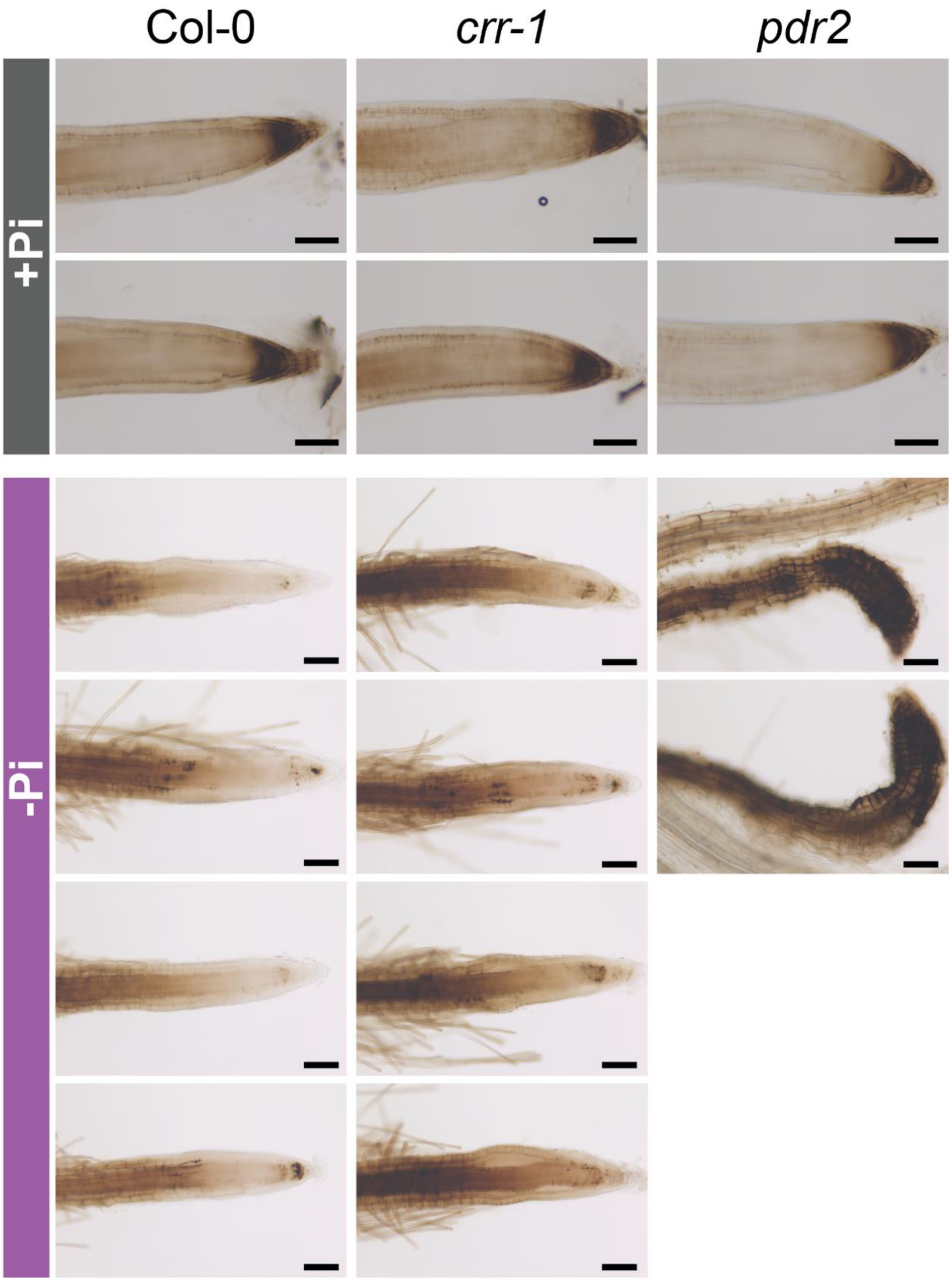

Supplementary Fig. 11 Perls-DAB staining of Col-0, *crr-1*, and *pdr2* root tips.
Images showing the variability of iron detection in the root meristem of the indicated genotypes grown for 7 days under plus (+Pi) and minus (-Pi) conditions. Scale = 100 µm.

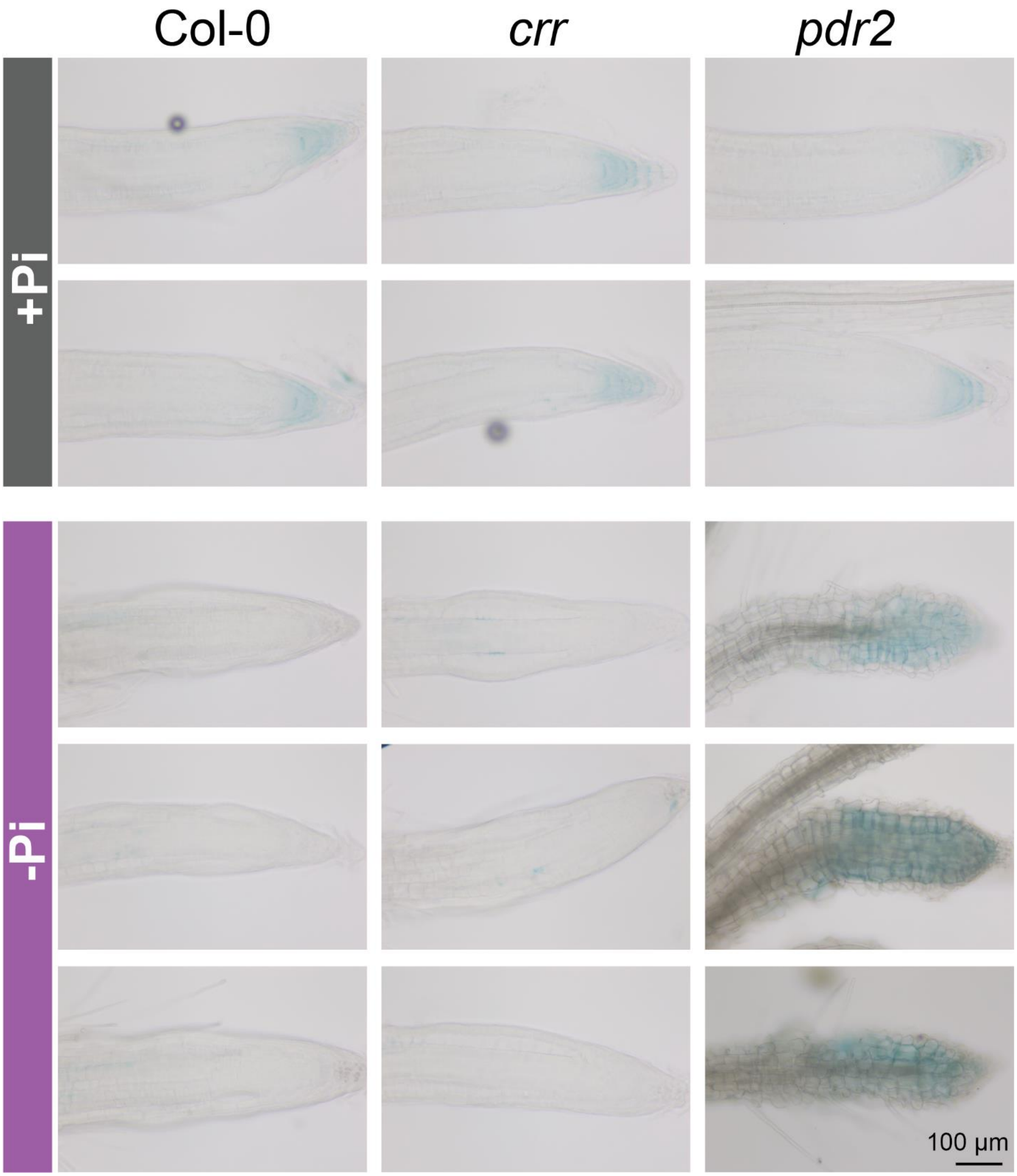

Supplementary Fig. 12 Perls staining of Col-0, *crr-1*, and *pdr2* root tips.
Images showing the variability of iron detection without DAB intensification in the root meristem of the indicated genotypes grown for 7 days under plus (+Pi) and minus (-Pi) conditions. Scale = 100 µm.

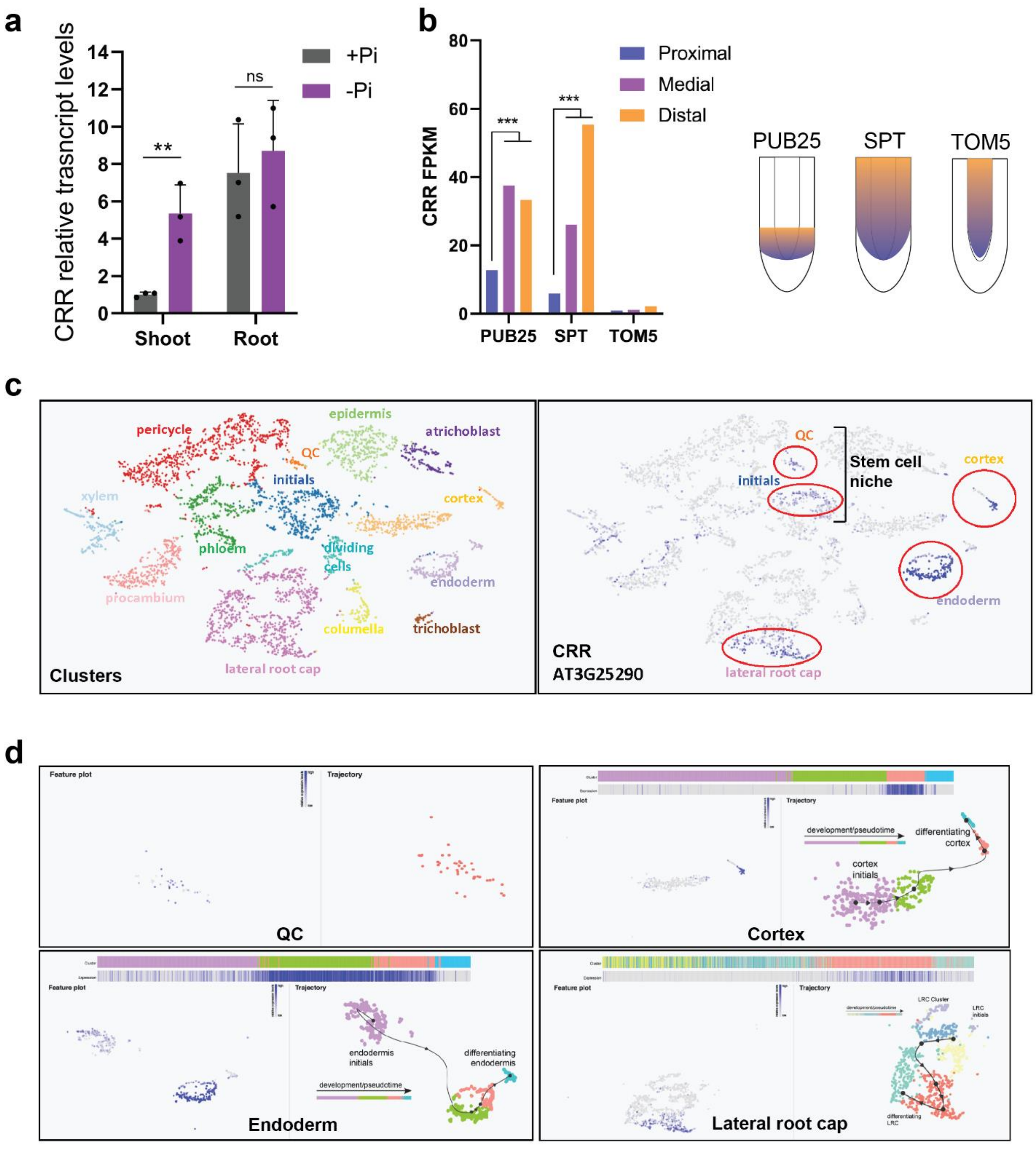

Supplementary Fig. 13 CRR expression pattern.

a, *CRR* is induced by phosphate starvation in shoots. Graph showing CRR relative transcripts levels in shoots and roots of plants grown for 7 days in plus (+Pi) and minus (-Pi) phosphate. *ACT2* was used as reference gene and data was further normalized to the shoot +Pi condition. Data is represented as the mean of 3 biological replicates ± s.d. For each tissue significant differences between +Pi and -Pi were analyzed using an unpaired two-tailed t-test (**P < 0.01). The P-value for the shoot comparison was 0,0084 and for roots 0,6158. The n=3 in all cases. **b**, CRR is expressed in the proximal root apical meristem. Data retrieved from Wendrich et al., 2017 (DOI: 10.1073/pnas.1707400114) showing *CRR*

expression levels in root cell populations which express the *PUB25*, *SPT* or *TOM5* gene. The scheme indicates the expression pattern of these 3 genes as well as the intensity of the expression along the root. **c**, Single-cell transcriptomic data from Arabidopsis root tips was retrieved from the Plant sc-Atlas (https://bioit3.irc.ugent.be/plant-sc-atlas/). The left panel shows a color-coded tSNE plot with the classification of sequenced cells into the different cell identities, and the right panel shows which cells express *CRR*. **d**, Single-cell transcriptomic data from Arabidopsis roots showing the expression of the *CRR* gene in QC, endodermis, cortex and lateral root cap cells in relation to the developmental trajectories of the same cells.

a

Promoter name	Id Gene	Cell type	Reference
LPR1	At1G23010	Stem cell niche (SCN), endodermis and provascular tissues	Muller et al., 2015. doi: 10.1016/j.devcel.2015.02.007
SCR	At3g54220	Root endodermis, quiescent center	Mustroph et al., 2009. doi: 10.1073/pnas.0906131106
SHR	At4g37650	Root vasculature	Mustroph et al., 2009. doi: 10.1073/pnas.0906131106
MYB36	At5G57620	Endodermis elongation	Pengxue Li et al., 2018. doi: 10.1016/j.cub.2018.07.028
WOL	At2g01830	Root vasculature	Mustroph et al., 2009. doi: 10.1073/pnas.0906131106
CO2	At1g62500	Root cortex meristematic zone	Mustroph et al., 2009. doi: 10.1073/pnas.0906131106
PEP	At1g09750	Root cortex elongation and maturation zone	Mustroph et al., 2009. doi: 10.1073/pnas.0906131106
ATL75	At1G49200	Pericycle	Denyer et al., 2019. doi: 10.1016/j.devcel.2019.02.022
LOVE1	AT5G20045	Root cap	Berhin et al., 2019. doi: 10.1016/j.cell.2019.01.005
WOX5	At3G11260	Quiescent center (QC)	Burkart et al., 2022. doi: 10.15252/embr.202154105

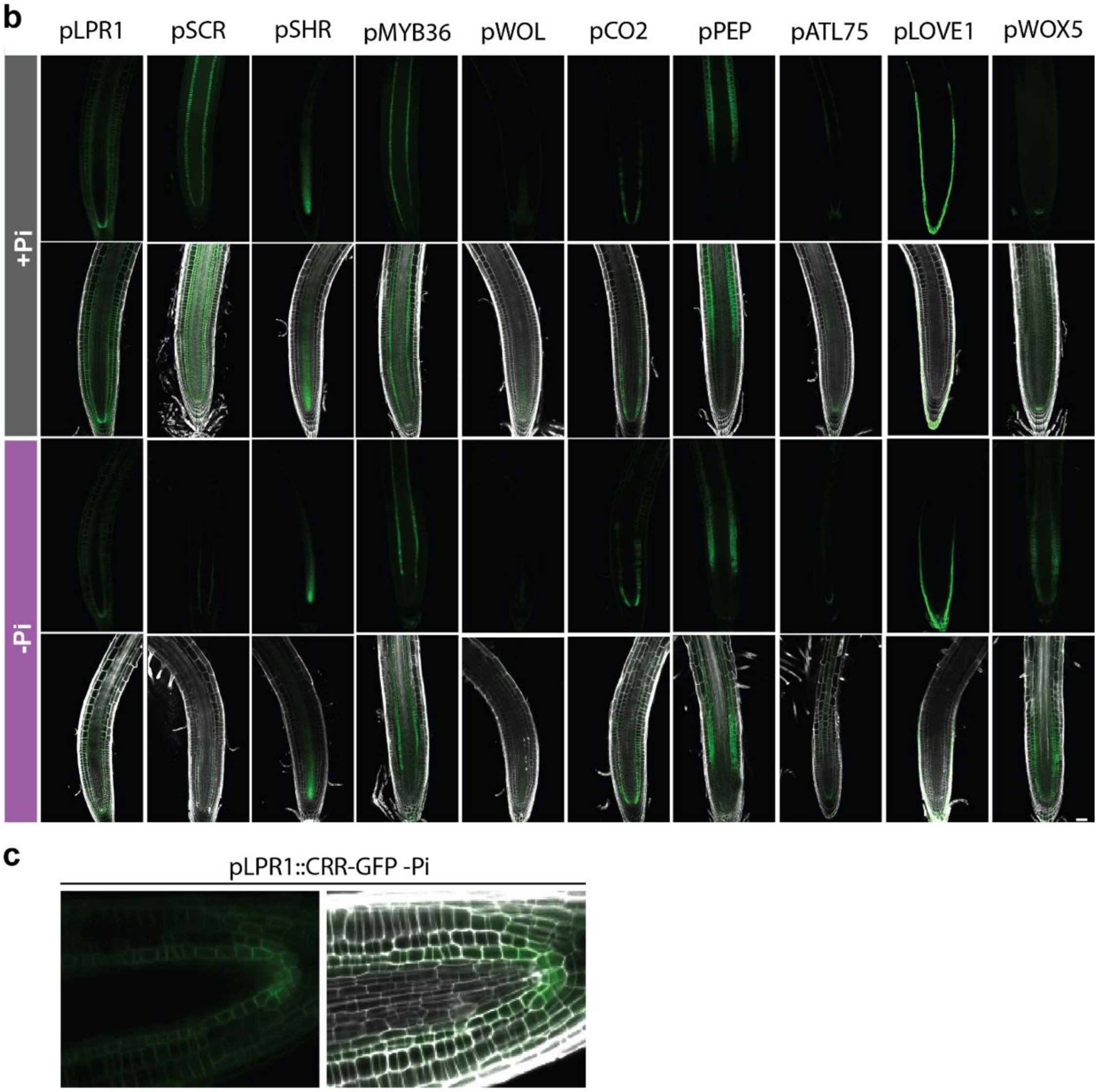

Supplementary Fig. 14 *CRR* expression pattern driven by different promotors.
a, Description of the promoters used to drive CRR-GFP expression. **b**, Confocal microscopy images of *crr* mutant roots showing the expression of CRR-GFP translational fusion under the control of different promotors. **c**, Close-up showing the expression of CRR-GFP under the control of LPR1 promoter under phosphate deficiency (-Pi).

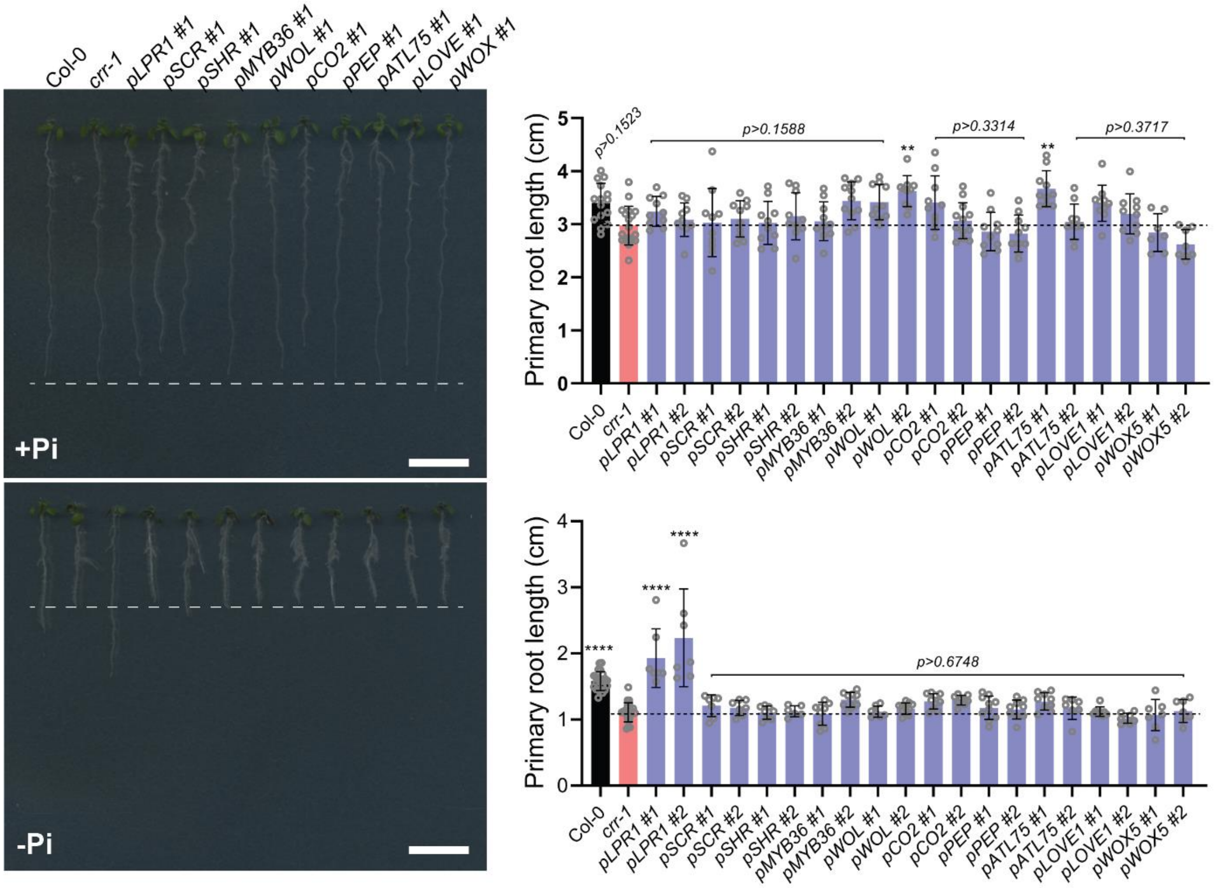

Supplementary Fig. 15 *CRR* expression driven by *LPR1* promoter complements *crr* mutant phenotype.

Root phenotype and root length quantification of Col-0, *crr-1*, and different *crr-1* mutant lines complemented with a CRR-GFP translational fusion under the control of the indicated promoters. Plants were grown for 7 days under plus (+Pi) or minus (-Pi) phosphate media and the root length of two independent lines showing the expected CRR-GFP expression pattern was quantified. The pictures show representative root phenotypes of one of the generated lines. Statistical analysis was performed using a One-way ANOVA followed by a Tukey's test. Significant differences against *crr-1* are indicated (****$P < 0.0001$; ***$P < 0.001$; **$P < 0.01$; *$P < 0.05$). Source data, including n and p values, are provided as a Source Data file.

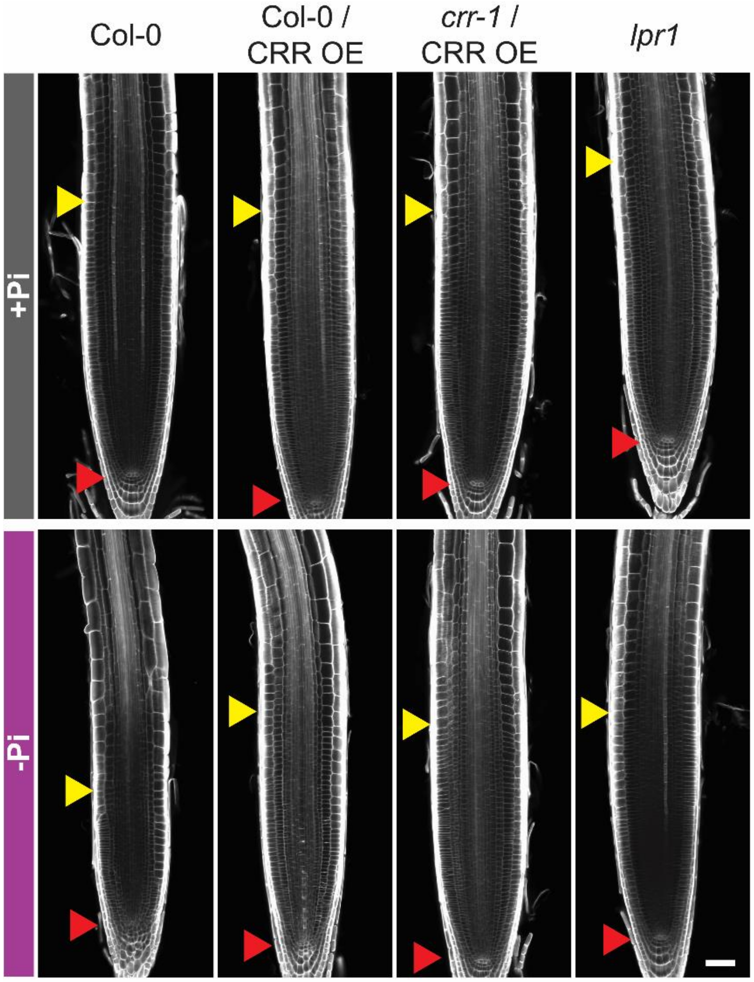

Supplementary Fig. 16 The root apical meristem of CRR OE lines is insensitive to phosphate starvation.

Confocal microscopy images showing the size and morphology of the root apical meristem of Col-0, CRR over-expressing lines (CRR OE) in a Col-0 or *crr-1* mutant background and *lpr1* seedlings grown in plus (+Pi) or minus (-Pi) phosphate conditions. Cell walls were stained using Calcofluor white. The quiescent center and the end of the meristem are indicated by red and yellow triangles, respectively. Scale: 20 µm.

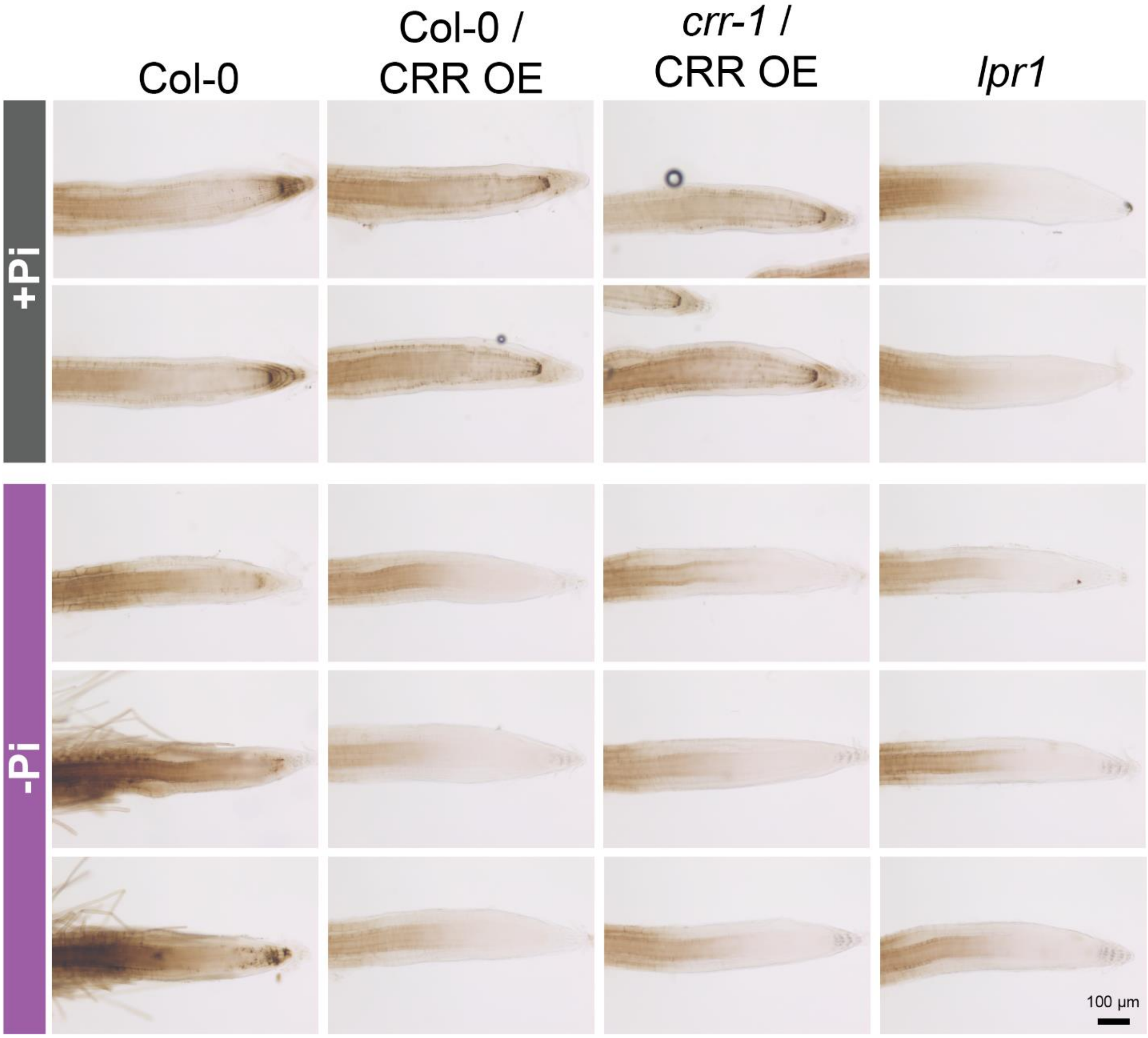

Supplementary Fig. 17 Perls-DAB staining of Col-0, *CRR* over-expressing lines, and *lpr1* root tips. Images showing the variability of iron detection in the root meristem of the indicated genotypes grown for 7 days under plus (+Pi) and minus (-Pi) conditions. Scale = 100 µm.

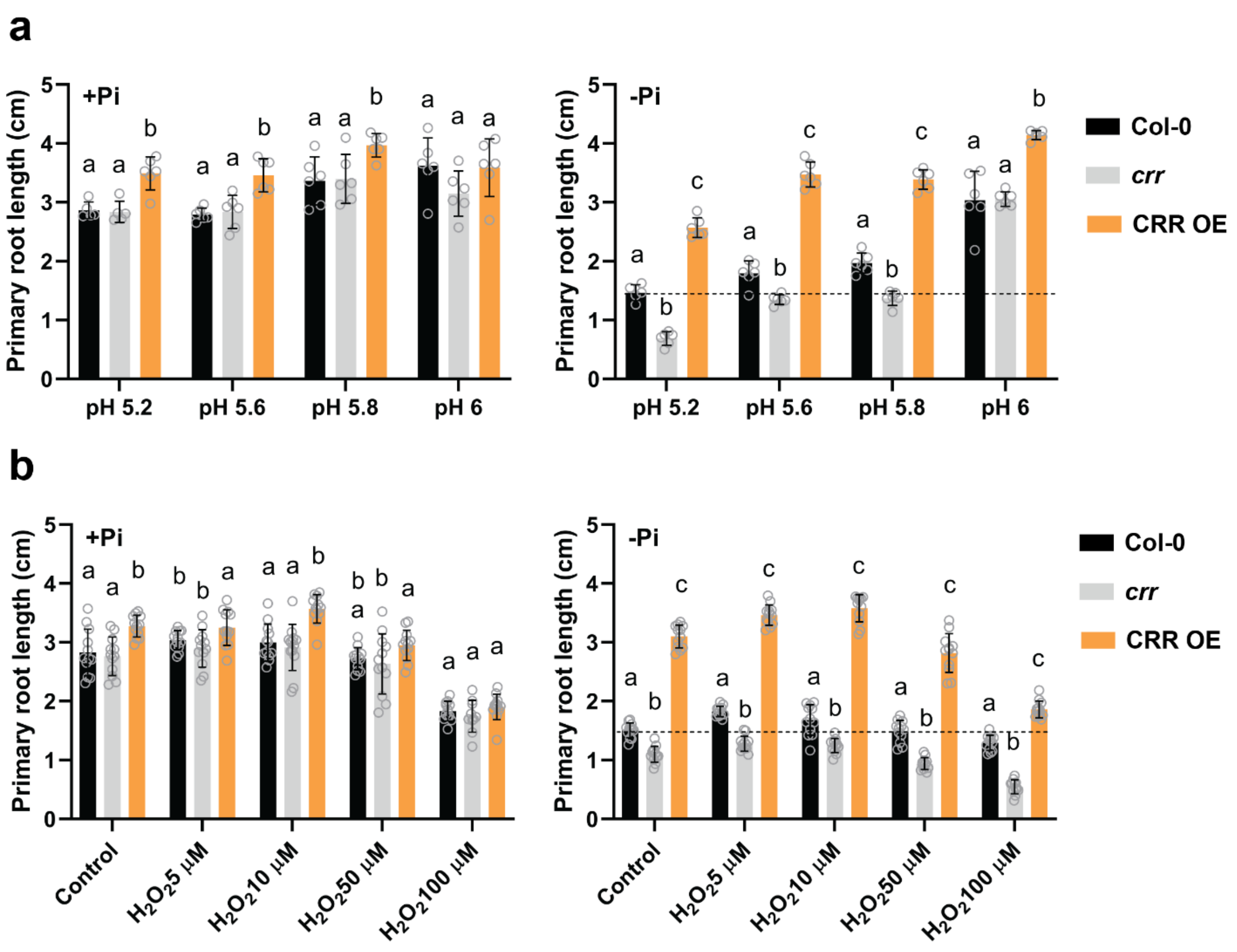

Supplementary Fig. 18 *crr* mutant phenotype is enhanced by low pH and hydrogen peroxide.
a, Primary root length of Col-0, *crr-1* and CRR OE of plants grown in plus (+Pi) or minus (-Pi) phosphate at different pH. **b**, Primary root length of the same Arabidopsis genotypes under increasing concentrations of hydrogen peroxide (H_2O_2). In (a) and (b) the data is represented as mean± s.d. (sample size is shown by circles). A One-way ANOVA followed by a Tukey's multiple comparisons test was used to assess the statistical differences. Significant differences ($P < 0.05$) within each particular condition are indicated by different letters. Source data, including n and p values, are provided as a Source Data file.

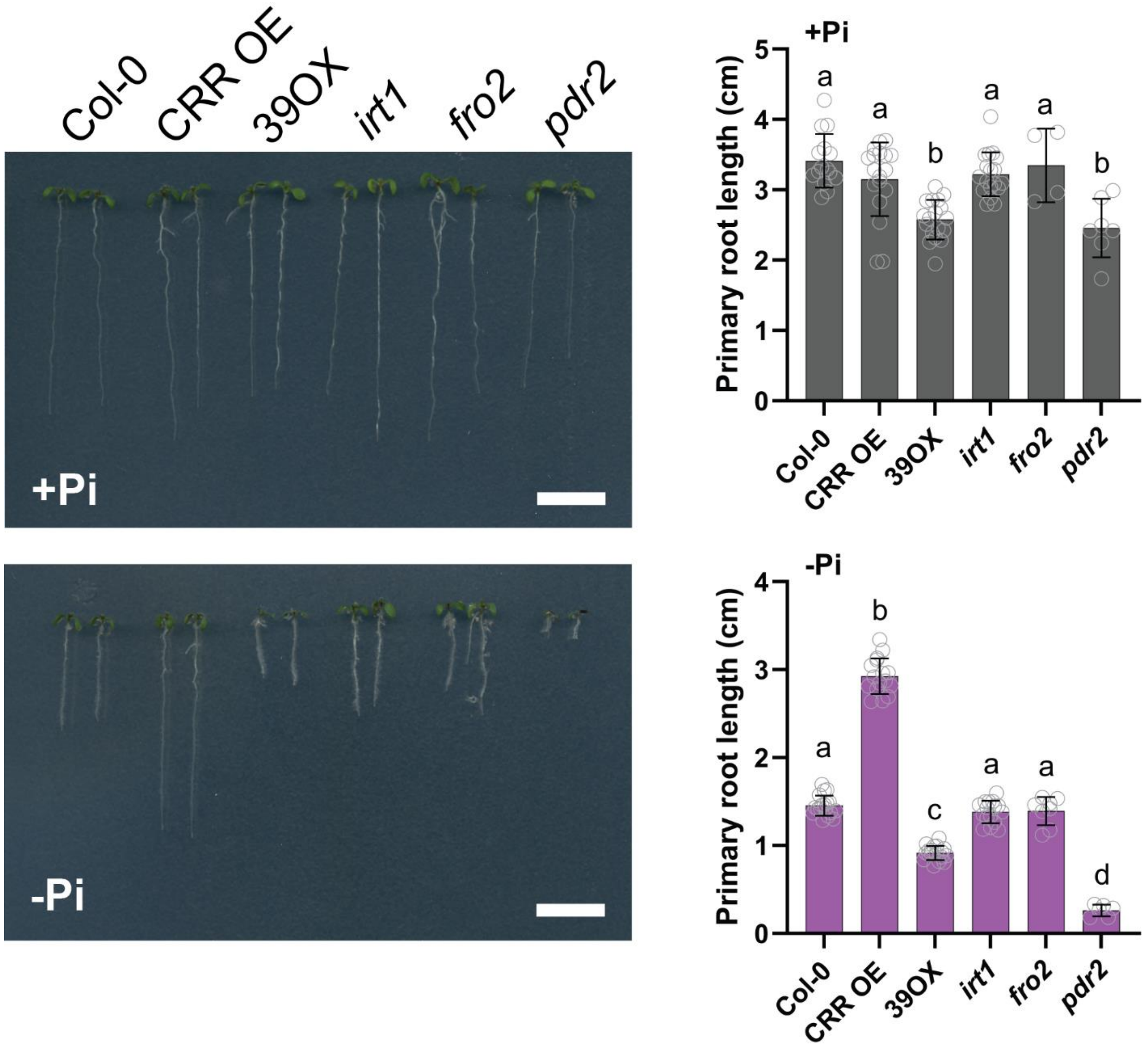

Supplementary Fig. 19 Primary root growth of the iron homeostasis mutants *irt1* and *fro2* under Pi deficiency are similar to Col-0.

Photographs showing Col-0, CRR OE, 390X, *irt1*, *fro2* and *pdr2* seedlings grown for 7 days under plus (+Pi) or minus (-P) phosphate conditions. Line 390X over-expresses the transcription factor bHIH39 and shows a strong increase in root ferric-reductase activity. Scale: 1 cm. Root length was quantified, and the statistical differences were assessed by a One-way ANOVA followed by a Tukey's test. Different letters indicate significant differences with a P-value < 0.05 and circles the sample size. Source data, including n and p values, are provided as a Source Data file.

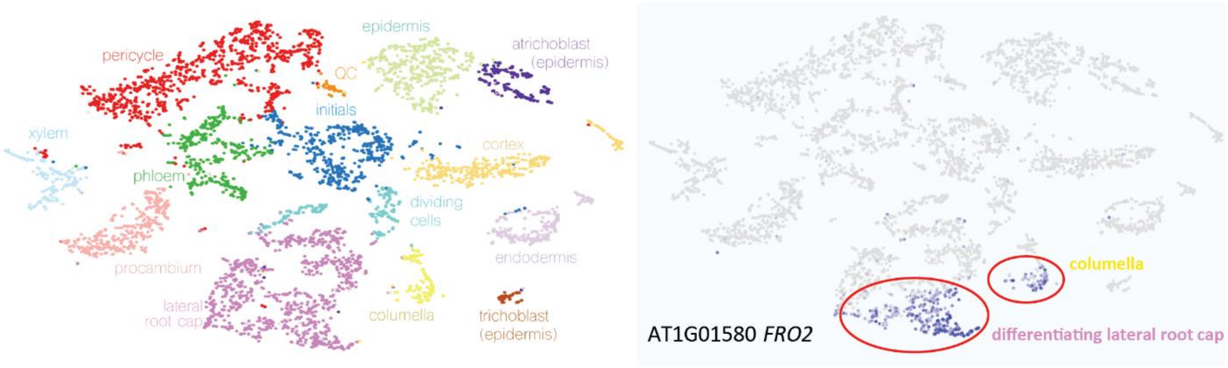

Supplementary Fig. 20 *FRO2* expression pattern in root tips.
Single-cell transcriptomic data from Arabidopsis root tips was retrieved from the Plant sc-Atlas (https://bioit3.irc.ugent.be/plant-sc-atlas/). The left panel shows a color-coded tSNE plot with the classification of sequenced cells into the different cell identities, and the right panel shows which cells express *FRO2*.

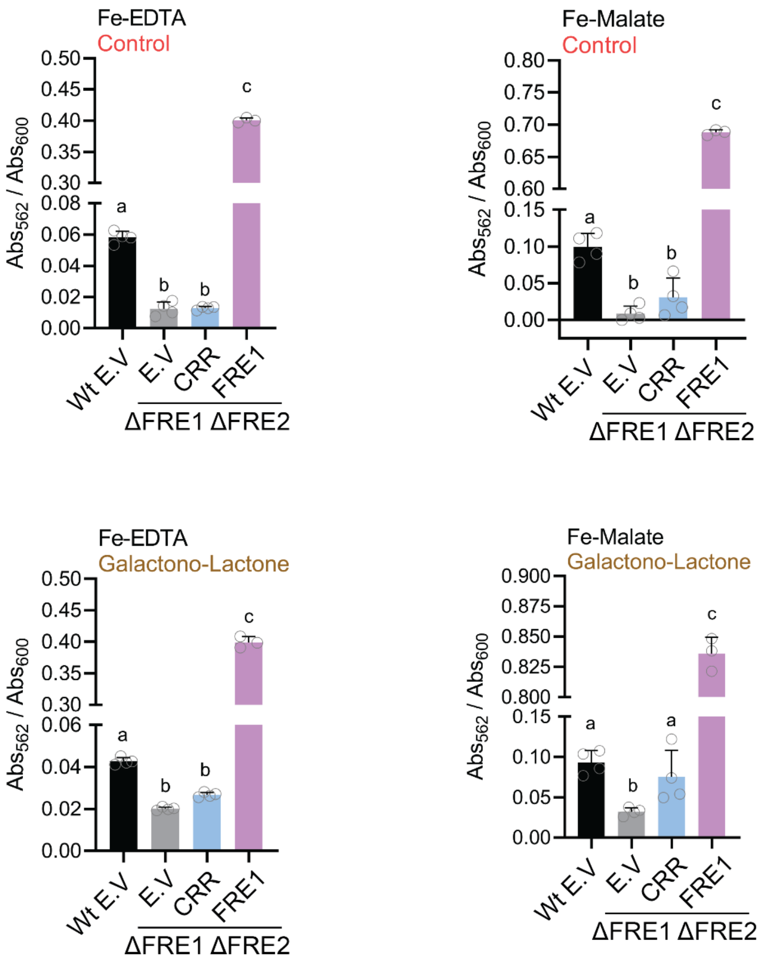

Supplementary Fig. 21 Ferrireductase assay in yeast using Fe(III)-EDTA and Fe(III)-Malate as electron acceptors.

The *Saccharomyces cerevisiae* strain S288C or a mutant lacking the two main yeast ferric reductases FRE1 and FRE2 (*Δfre1 Δfre2*) were transformed with a plasmid driving the expression of *CRR* or *FRE1* under the control of the strong constitutive promotor *GPD*. The wild-type (WT) strain was transformed with an empty vector as a control (EV). All the strains were grown in synthetic defined (SD) media as control or SD supplemented with the ascorbate biosynthetic precursor galactono1,4-lactone. Either Fe(III)-EDTA or Fe(III)-Malate (both at 500 µM) were used as electron acceptors and iron reduction was quantified using the ferrozine method. The production of Fe^{2+}-ferrozine complexes were determined by reading the absorbance at 562 nm and normalized by cell concentration determined as the OD600. The statistical differences were analyzed using a one-way ANOVA followed by Tukey's multiple comparisons test. Different letters indicate significant differences with a P-value <0.05. Data are presented as means ± sd and the sample size is indicated by circles. Source data, including n and p values, are provided as a Source Data file.

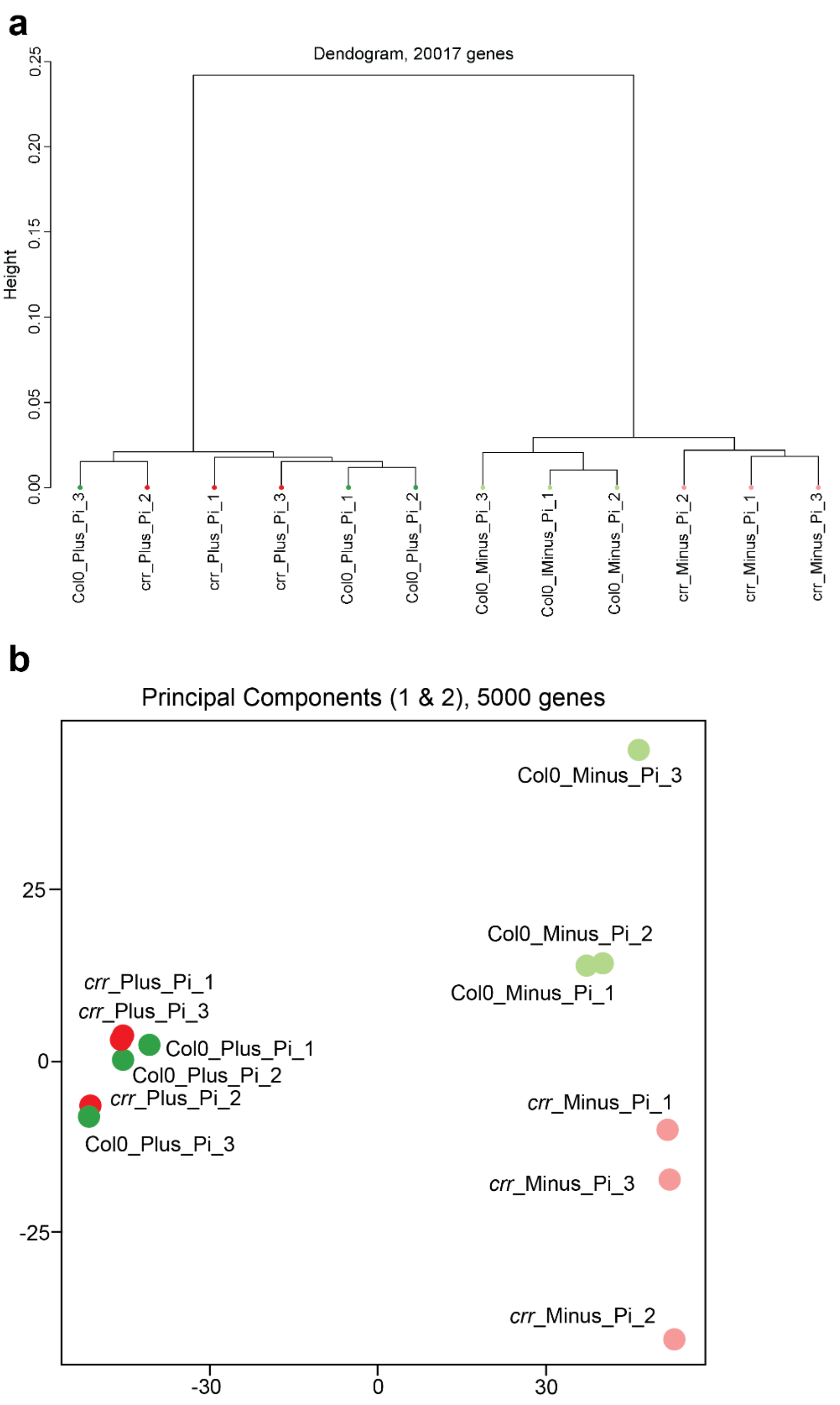

Supplementary Fig. 22 Quality control and principal component analysis of the Col-0 and *crr-1* RNA-seq samples used in the transcriptomic analysis.

a, Dendrogram showing the clustering of the 3 biological replicates used in the RNA-seq analysis. **b**, Principal component analysis (PCA) based on the expression of the most 5000 variable genes. The PCA shows that Col-0 and *crr-1* transcriptomes cluster together in plus phosphate (+Pi) conditions, while they diverge under minus phosphate (-Pi) conditions.

a

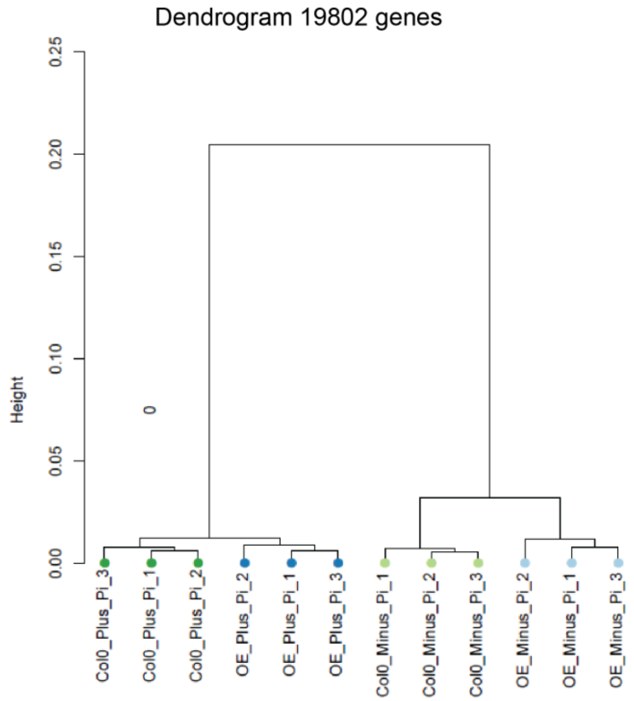

b

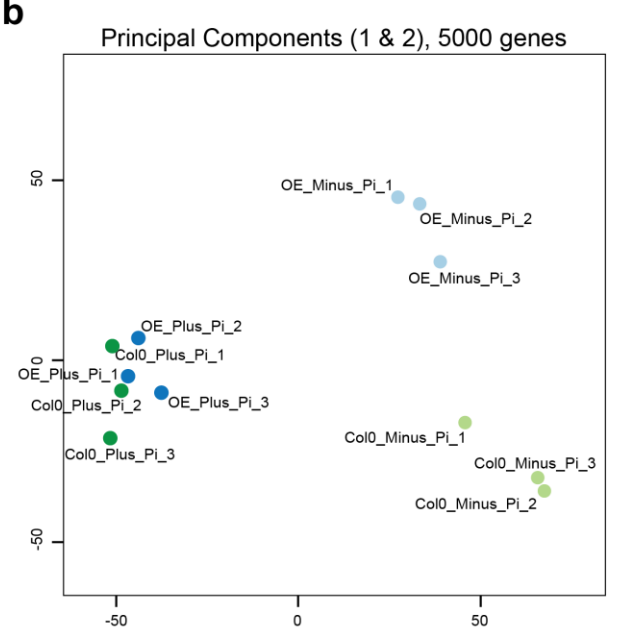

Supplementary Fig. 23 Quality control and principal component analysis of the Col-0 and CRR OE RNA-seq samples used in the transcriptomic analysis.

a, Dendrogram showing that in all conditions the 3 biological replicates used in the RNA-seq analysis cluster together. **b**, Principal component analysis (PCA) based on the expression of the most 5000 variable genes. The PCA shows that Col-0 and CRR OE transcriptomes cluster together in plus phosphate (+Pi) conditions, while they diverge under minus phosphate (-Pi) conditions.

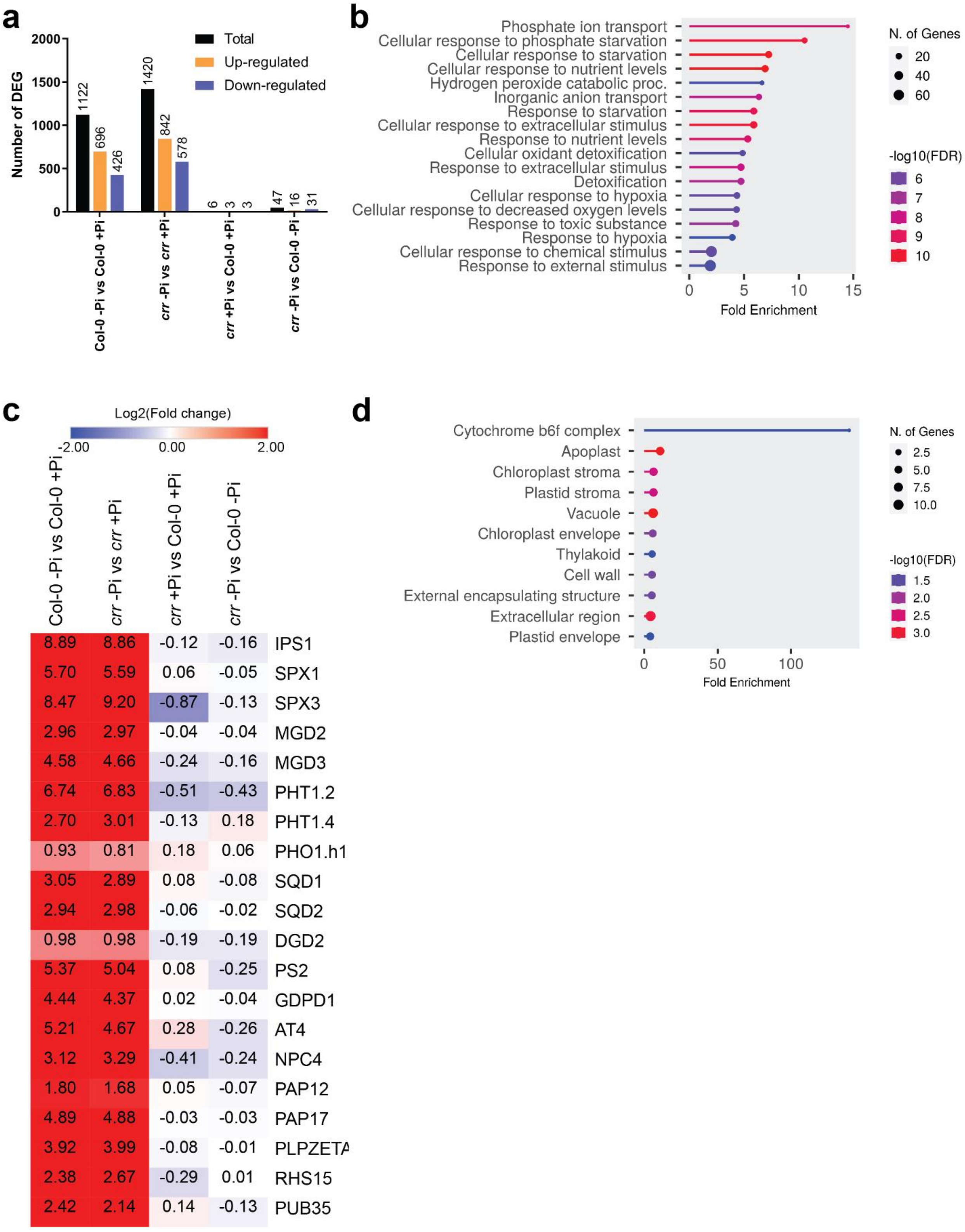
a
Number of DEG
2000
1500
1000
500
0
Total
Up-regulated
Down-regulated
1122
696
426
1420
842
578
6
3
3
47
16
31
Col-0 -Pi vs Col-0 +Pi
crr -Pi vs crr +Pi
crr +Pi vs Col-0 +Pi
crr -Pi vs Col-0 -Pi
b
Phosphate ion transport
Cellular response to phosphate starvation
Cellular response to starvation
Cellular response to nutrient levels
Hydrogen peroxide catabolic proc.
Inorganic anion transport
Response to starvation
Cellular response to extracellular stimulus
Response to nutrient levels
Cellular oxidant detoxification
Response to extracellular stimulus
Detoxification
Cellular response to hypoxia
Cellular response to decreased oxygen levels
Response to toxic substance
Response to hypoxia
Cellular response to chemical stimulus
Response to external stimulus
0
5
10
15
Fold Enrichment
N. of Genes
20
40
60
-log10(FDR)
6
7
8
9
10
c
Log2(Fold change)
-2.00
0.00
2.00
Col-0 -Pi vs Col-0 +Pi
crr -Pi vs crr +Pi
crr +Pi vs Col-0 +Pi
crr -Pi vs Col-0 -Pi
8.89 8.86 -0.12 -0.16 IPS1
5.70 5.59 0.06 -0.05 SPX1
8.47 9.20 -0.87 -0.13 SPX3
2.96 2.97 -0.04 -0.04 MGD2
4.58 4.66 -0.24 -0.16 MGD3
6.74 6.83 -0.51 -0.43 PHT1.2
2.70 3.01 -0.13 0.18 PHT1.4
0.93 0.81 0.18 0.06 PHO1.h1
3.05 2.89 0.08 -0.08 SQD1
2.94 2.98 -0.06 -0.02 SQD2
0.98 0.98 -0.19 -0.19 DGD2
5.37 5.04 0.08 -0.25 PS2
4.44 4.37 0.02 -0.04 GDPD1
5.21 4.67 0.28 -0.26 AT4
3.12 3.29 -0.41 -0.24 NPC4
1.80 1.68 0.05 -0.07 PAP12
4.89 4.88 -0.03 -0.03 PAP17
3.92 3.99 -0.08 -0.01 PLPZETA
2.38 2.67 -0.29 0.01 RHS15
2.42 2.14 0.14 -0.13 PUB35
d
Cytochrome b6f complex
Apoplast
Chloroplast stroma
Plastid stroma
Vacuole
Chloroplast envelope
Thylakoid
Cell wall
External encapsulating structure
Extracellular region
Plastid envelope
0
50
100
Fold Enrichment
N. of Genes
2.5
5.0
7.5
10.0
-log10(FDR)
1.5
2.0
2.5
3.0

Supplementary Fig. 24 Analysis of the differential expressed genes between Col-0 and *crr-1* roots grown in +Pi and -Pi conditions.

a, Graph showing the total, up- and down-regulated number of differential expressed genes (DEG) found in the pairwise-comparisons indicated in the x-axis. **b**, Gene ontology (GO) enrichment analysis focused on molecular functions for DEG found the -Pi/+Pi treatments and shared by both Col-0 and *crr-1*. **c**, Heat map showing the expression pattern of canonical phosphate starvation responsive genes. Data is expressed as the log2 of the fold change. **d**, GO enrichment analysis focused on cellular compartment categories. The DEG found in the interaction between the genotype and treatment was used as input. In (b) and (d) The lollipop graph shows for each category the fold enrichment, the number of genes (N. of genes) and the -log10 of the fold discovery rate (FDR).

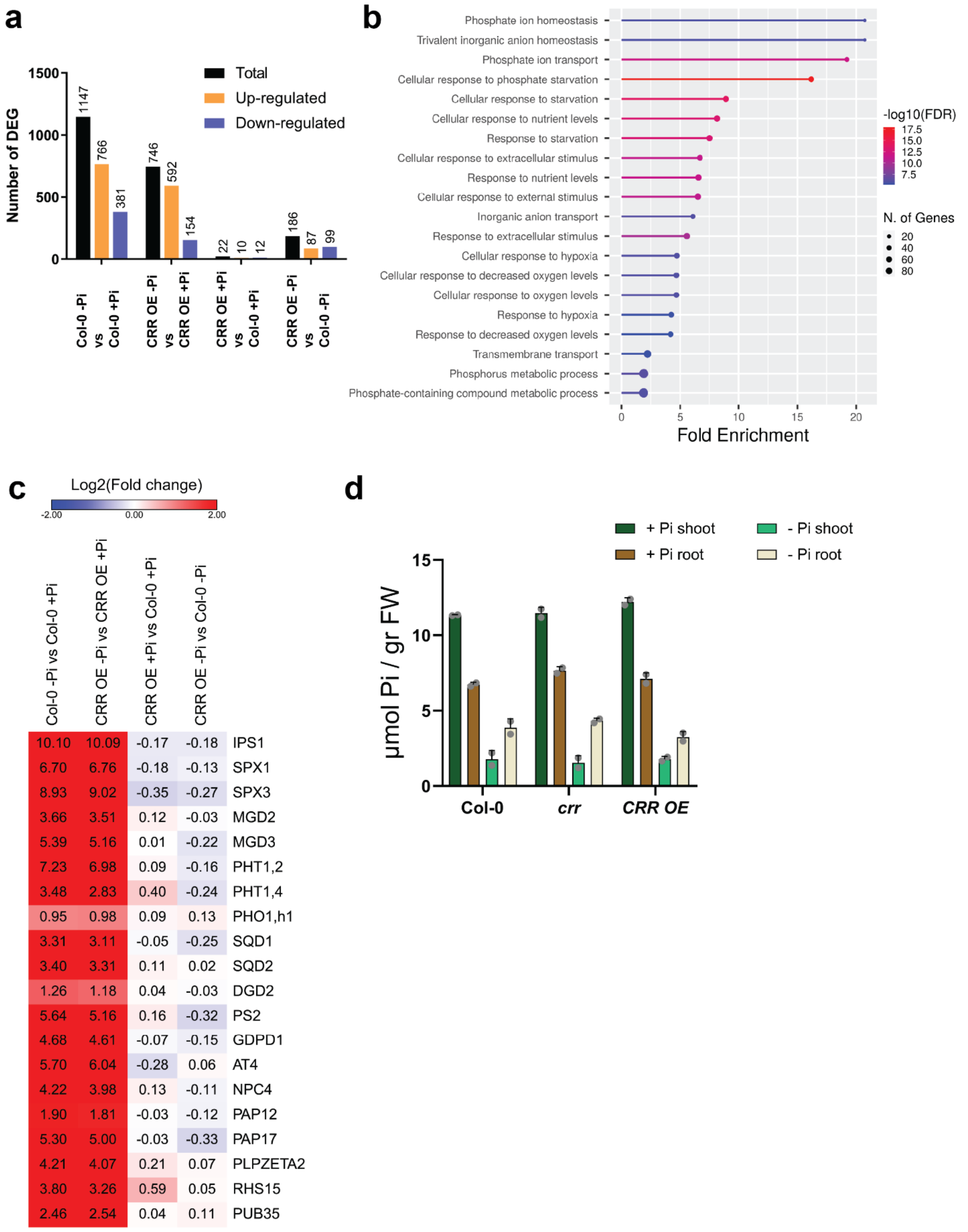
a
1500
1000
500
0
Number of DEG
Total
Up-regulated
Down-regulated
1147
766
381
746
592
154
22
10
12
186
87
99
Col-0 -Pi vs Col-0 +Pi
CRR OE -Pi vs CRR OE +Pi
CRR OE +Pi vs Col-0 +Pi
CRR OE -Pi vs Col-0 -Pi
b
Phosphate ion homeostasis
Trivalent inorganic anion homeostasis
Phosphate ion transport
Cellular response to phosphate starvation
Cellular response to starvation
Cellular response to nutrient levels
Response to starvation
Cellular response to extracellular stimulus
Response to nutrient levels
Cellular response to external stimulus
Inorganic anion transport
Response to extracellular stimulus
Cellular response to hypoxia
Cellular response to decreased oxygen levels
Cellular response to oxygen levels
Response to hypoxia
Response to decreased oxygen levels
Transmembrane transport
Phosphorus metabolic process
Phosphate-containing compound metabolic process
0
5
10
15
20
Fold Enrichment
-log10(FDR)
17.5
15.0
12.5
10.0
7.5
N. of Genes
20
40
60
80
c
Log2(Fold change)
-2.00
0.00
2.00
Col-0 -Pi vs Col-0 +Pi
CRR OE -Pi vs CRR OE +Pi
CRR OE +Pi vs Col-0 +Pi
CRR OE -Pi vs Col-0 -Pi
10.10 10.09 -0.17 -0.18 IPS1
6.70 6.76 -0.18 -0.13 SPX1
8.93 9.02 -0.35 -0.27 SPX3
3.66 3.51 0.12 -0.03 MGD2
5.39 5.16 0.01 -0.22 MGD3
7.23 6.98 0.09 -0.16 PHT1,2
3.48 2.83 0.40 -0.24 PHT1,4
0.95 0.98 0.09 0.13 PHO1,h1
3.31 3.11 -0.05 -0.25 SQD1
3.40 3.31 0.11 0.02 SQD2
1.26 1.18 0.04 -0.03 DGD2
5.64 5.16 0.16 -0.32 PS2
4.68 4.61 -0.07 -0.15 GDPD1
5.70 6.04 -0.28 0.06 AT4
4.22 3.98 0.13 -0.11 NPC4
1.90 1.81 -0.03 -0.12 PAP12
5.30 5.00 -0.03 -0.33 PAP17
4.21 4.07 0.21 0.07 PLPZETA2
3.80 3.26 0.59 0.05 RHS15
2.46 2.54 0.04 0.11 PUB35
d
+ Pi shoot
+ Pi root
- Pi shoot
- Pi root
15
10
5
0
µmol Pi / gr FW
Col-0
crr
CRR OE

Supplementary Fig. 25 Analysis of the differential expressed genes between Col-0 and CRR OE roots grown in +Pi and -Pi conditions.

a, Graph showing the total, up- and down-regulated number of differential expressed genes (DEG) found in the pairwise-comparisons indicated in the x-axis. **b**, Gene ontology (GO) enrichment analysis focused on molecular functions for DEG found the -Pi/+Pi treatments and shared by both Col-0 and CRR OE. The lollipop graph shows for each category the fold enrichment, the number of genes (N. of genes) and the -log10 of the fold discovery rate (FDR). **c**, Heat map showing the expression pattern of canonical phosphate starvation responsive genes. Data is expressed as the log2 of the fold change. **d**, Inorganic phosphate quantification of Col-0, *crr-1*, and CRR OE roots and shoots from seedlings grown in plus (+Pi) or minus (-Pi) phosphate conditions. Data represented as mean± s.d; n=2 in all cases. One-way ANOVAs analysis revealed no significant differences between the genotypes analyzed. Source data, including n and p values, are provided as a Source Data file.

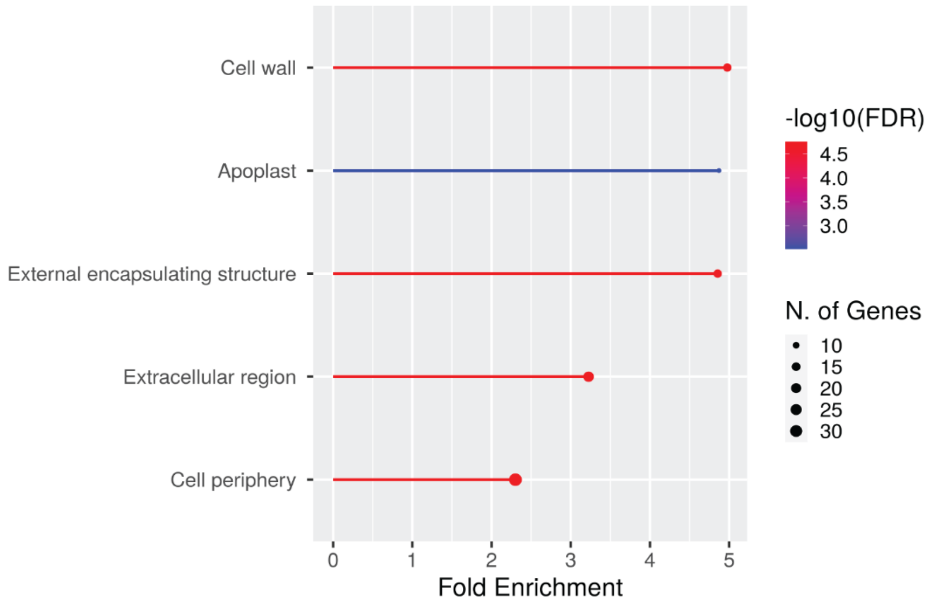

Supplementary Fig. 26 GO enrichment analysis of CRR OE differentially expressed genes (DEG), focused on cellular compartment categories.

The DEG found in the interaction between the genotype and treatment was used as input. The lollipop graph shows for each category the fold enrichment, the number of genes (N. of genes) and the -log10 of the FDR.

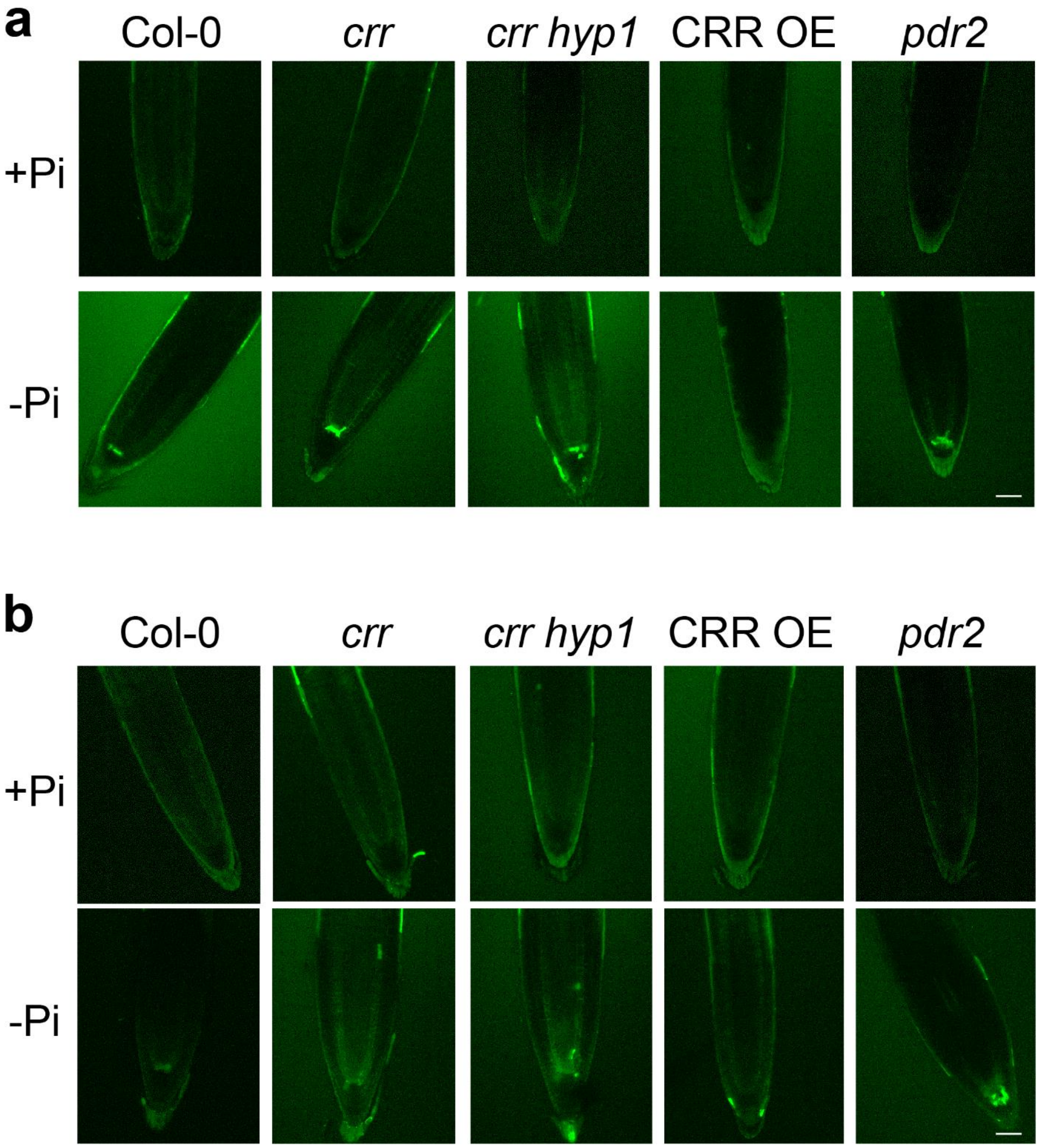

Supplementary Fig. 27 ROS production is enhanced in *crr-1* and *crr-1 hyp1* double mutant meristems under phosphate deficiency.

Confocal microscopy images showing ROS production in root tips of the indicated Arabidopsis genotypes. Plants were grown for 7 days under plus (+Pi) and minus (-Pi) phosphate conditions and roots were stained with the general ROS probe Carboxy-H2DCFDA (excitation 488 nm). Two independent experiments are shown, with laser intensity set at 2% (a) and 1% (b). Scale: 50 μM.

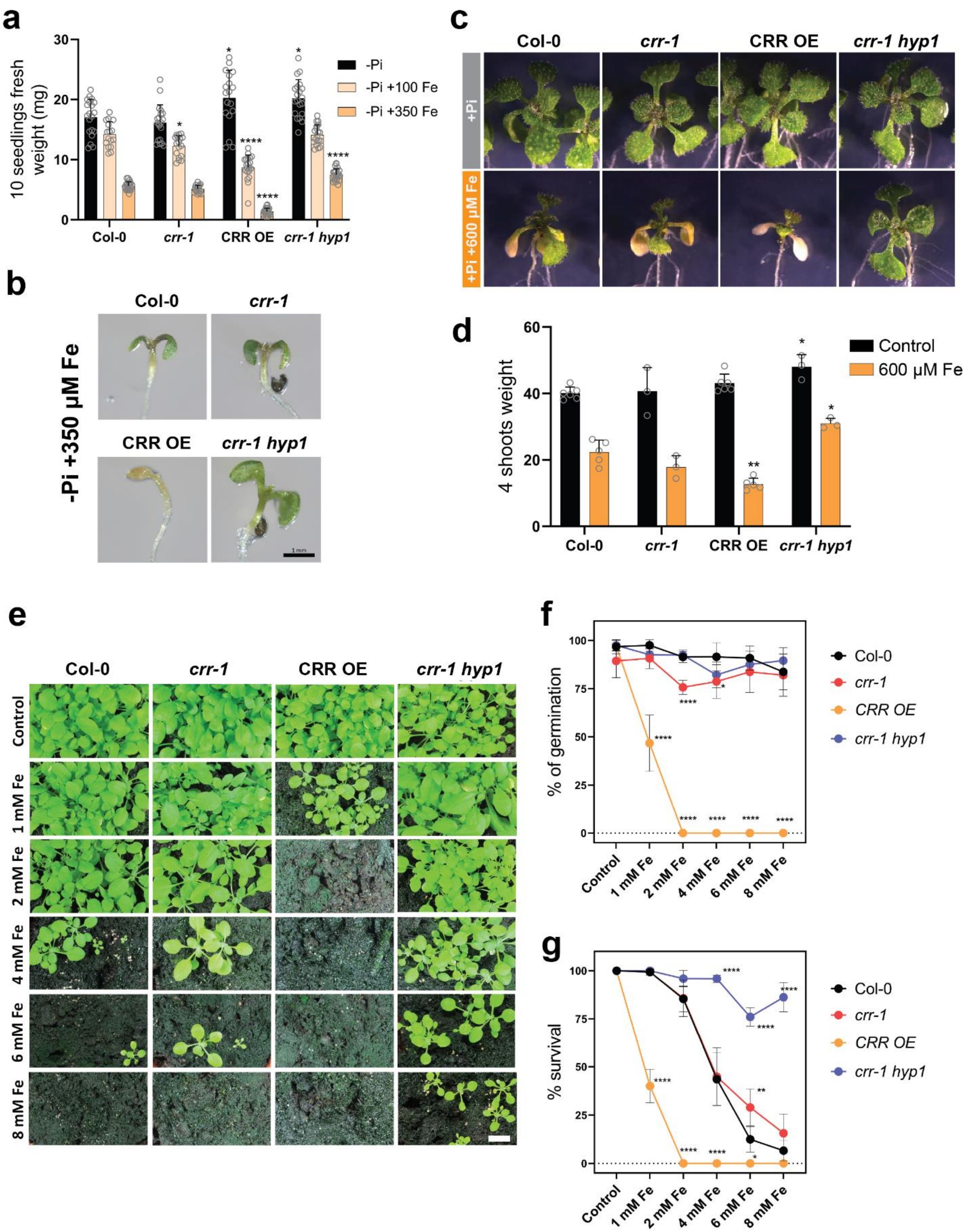
a
10 seedlings fresh weight (mg)
0
10
20
30
-Pi
-Pi +100 Fe
-Pi +350 Fe
Col-0
crr-1
CRR OE
crr-1 hyp1
b
-Pi +350 µM Fe
Col-0
crr-1
CRR OE
crr-1 hyp1
1 mm
c
Col-0
crr-1
CRR OE
crr-1 hyp1
+Pi
+Pi +600 µM Fe
d
4 shoots weight
0
20
40
60
Control
600 µM Fe
Col-0
crr-1
CRR OE
crr-1 hyp1
e
Col-0
crr-1
CRR OE
crr-1 hyp1
Control
1 mM Fe
2 mM Fe
4 mM Fe
6 mM Fe
8 mM Fe
f
% of germination
0
25
50
75
100
Control
1 mM Fe
2 mM Fe
4 mM Fe
6 mM Fe
8 mM Fe
Col-0
crr-1
CRR OE
crr-1 hyp1
g
% survival
0
25
50
75
100
Control
1 mM Fe
2 mM Fe
4 mM Fe
6 mM Fe
8 mM Fe
Col-0
crr-1
CRR OE
crr-1 hyp1

Supplementary Fig. 28 The CYBDOM proteins CRR and HYP1 are involved in iron toxicity tolerance.
a, Effect of iron toxicity under phosphate starvation conditions. Col-0, *crr-1*, CRR OE and *crr-1 hyp1* double mutant were grown in minus phosphate (-Pi) conditions with the addition of increasing amounts of Fe-EDTA. Seven days after germination the fresh weight of 10 seedlings was recorded. **b**, Representative images showing the shoot phenotypes of seedlings grown in -Pi and 350 µM of Fe-EDTA. **c**, Effect of high iron levels on 7 days old seedlings. Plants were grown for 7 days in +Pi and then transferred to plates containing either the same media as a control or additional iron (600 µM Fe-EDTA). Four days after being transferred the shoots were photographed. **d**, Quantification of shoot biomass from the plants described in (c), expressed as the weight of 4 shoots (mg). In (a) and (d) data is represented as mean ± s.d (sample size is indicated by circles) and the statistical differences were analyzed using a One-way ANOVA followed by a Tukey's multiple comparisons test. Significant differences against Col-0 are indicated (***P < 0.001; **P < 0.01; *P < 0.05). **e**, Effect of iron toxicity on plants grown in soil. Seeds were sown in soil watered with tap water supplemented with increasing concentrations of Fe-EDTA. After 15 days the pots were photographed. Scale: 1 cm. **f**, Percentage of seeds that germinated in five days after treatment. **g**, For the plants in (f) that had germinated by day 5, the percentage of plants that remained green and continued to develop for an additional 10 days was quantified. In (f) and (g), the data are the means ± sd (n=5). The statistical differences within each condition were analyzed using a one-way ANOVA followed by Tukey's multiple comparisons test. Significant differences from Col-0 are indicated (***$P < 0.001$; **$P < 0.01$; *$P < 0.05$). Source data, including n and p values, are provided as a Source Data file.

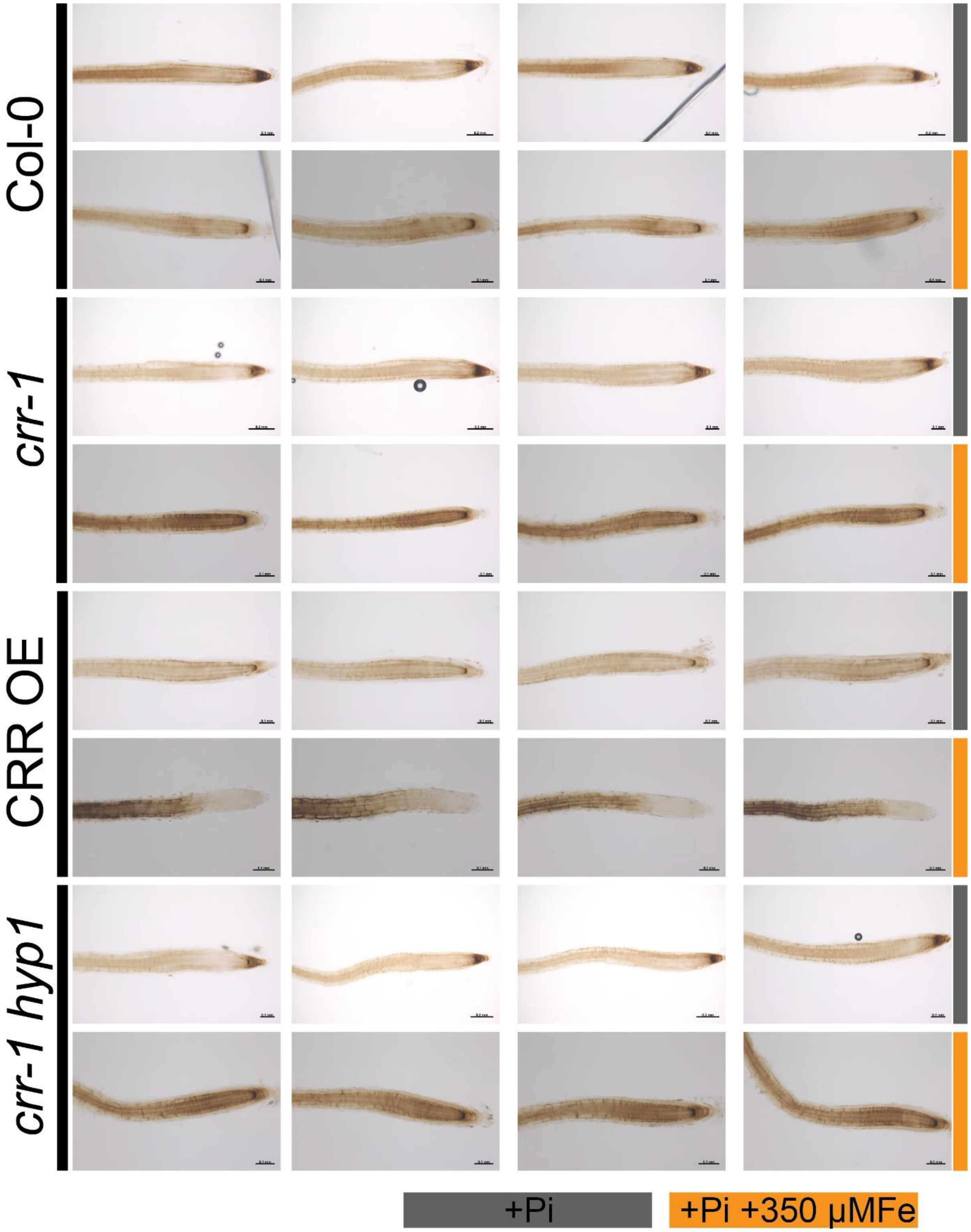

Supplementary Fig. 29 Perls-DAB staining of root tips under Fe stress.
Images showing Perls-DAB staining of root tips from plants grown for 7 days under plus (+Pi) phosphate conditions or under the same media supplemented with Fe(III)-EDTA (+Pi + 350 µM Fe). Scale: 0.1 mm.

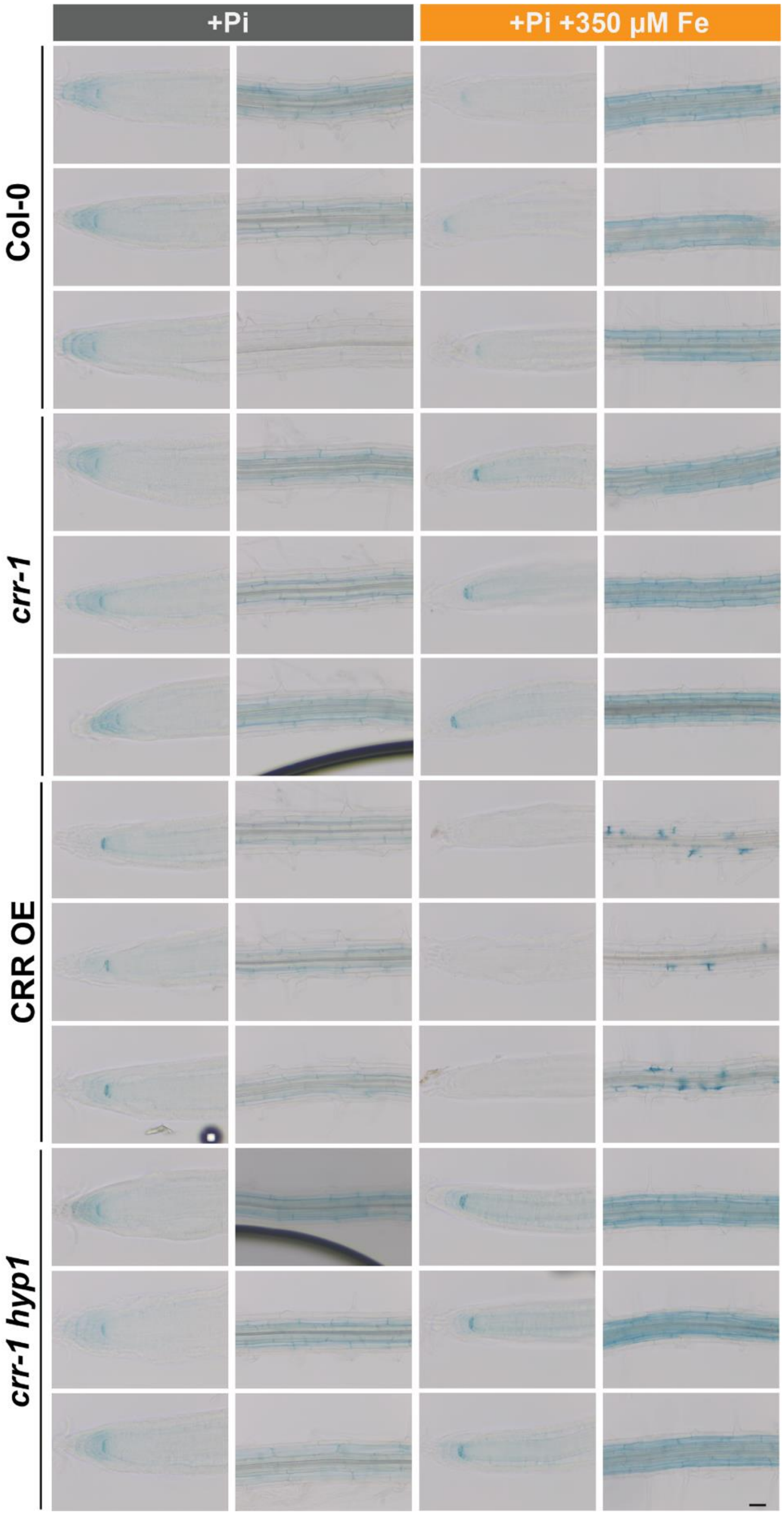

Supplementary Fig. 30 Perls staining of root tips under Fe stress.

Images showing Perls staining of root tips from plants grown for 7 days under plus (+Pi) phosphate conditions or under the same media supplemented with Fe(III)-EDTA (+Pi + 350 µM Fe). Scale: 50 µM.

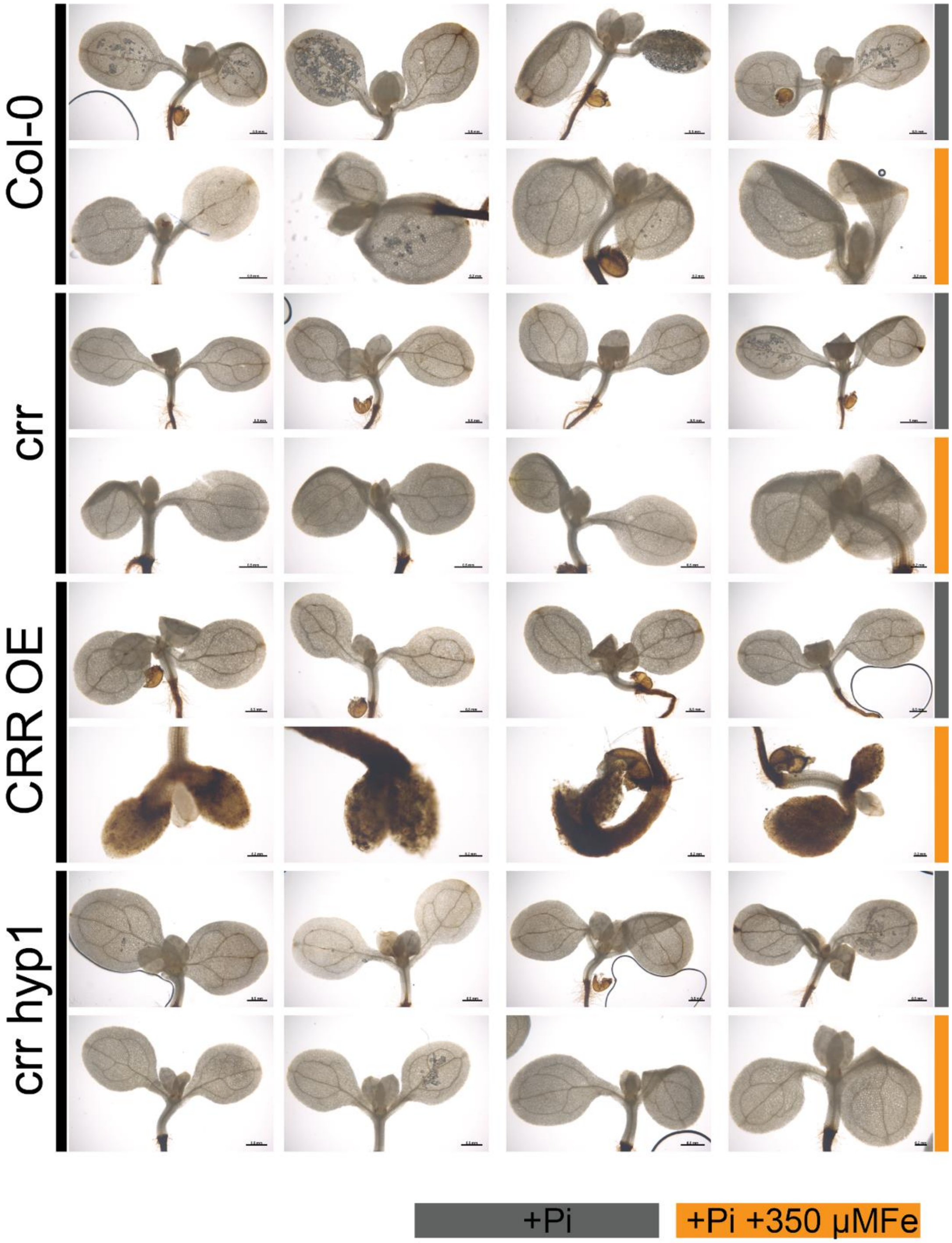

Supplementary Fig. 31 Perls-DAB staining of cotyledons under Fe stress.
Plants growing for 7 days under plus (+Pi) phosphate conditions or under the same media supplemented with Fe(III)-EDTA (+Pi + 350 µM Fe), were subjected to Perls-DAB staining. Four representative images are shown per genotype and condition.

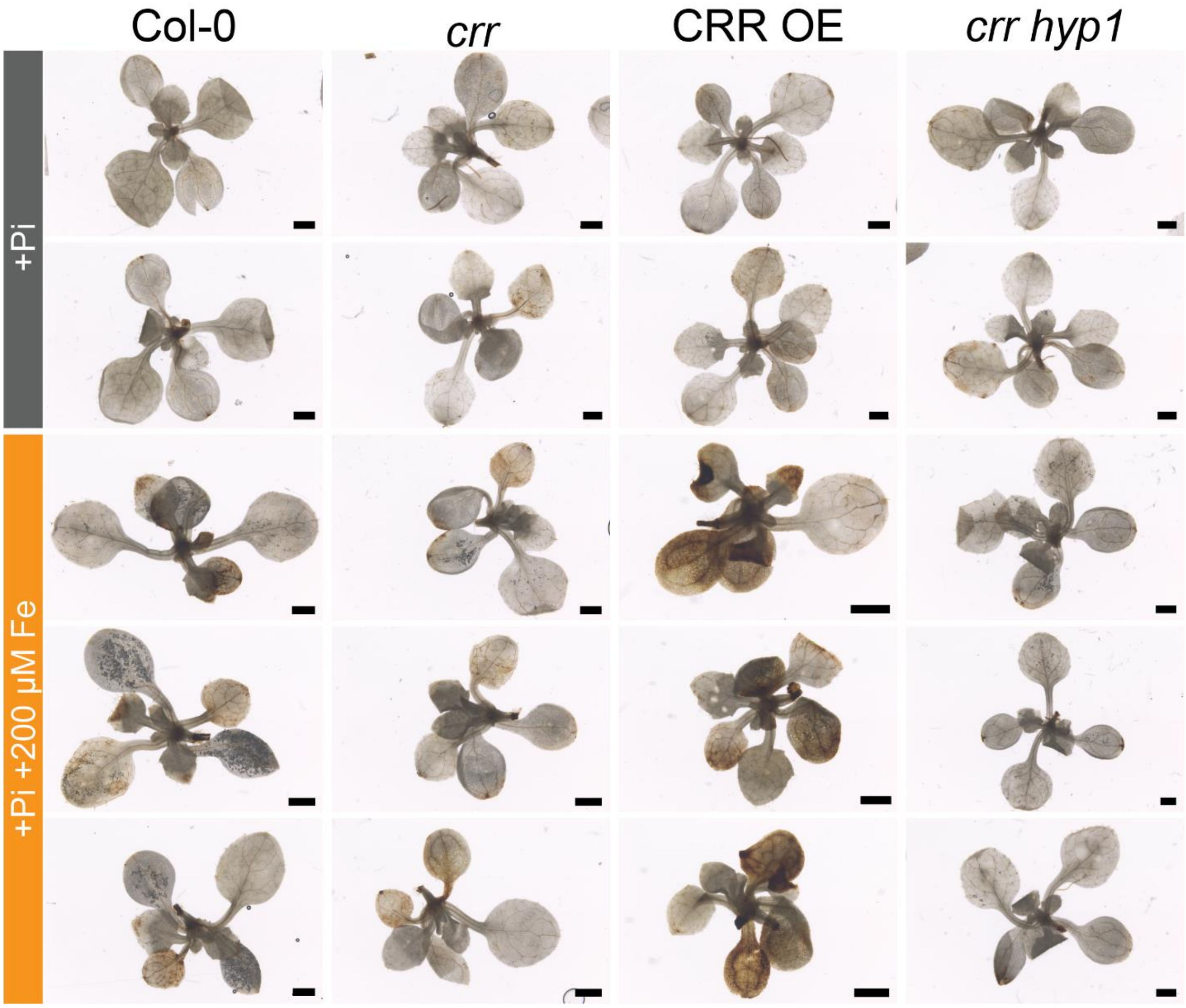

Supplementary Fig. 32 CRR OE line over-accumulates Fe in true leaves under iron stress. Images show Perls-DAB staining of cotyledons and true leaves from Arabidopsis seedlings grown in control conditions (+Pi) or under iron stress. Col-0, *crr-1*, CRR OE, and *crr-1* hyp1 plants were grown either for 11 days in +Pi or +Pi +200 µM Fe (a very mild iron stress). The scale bar represents 1 mm.

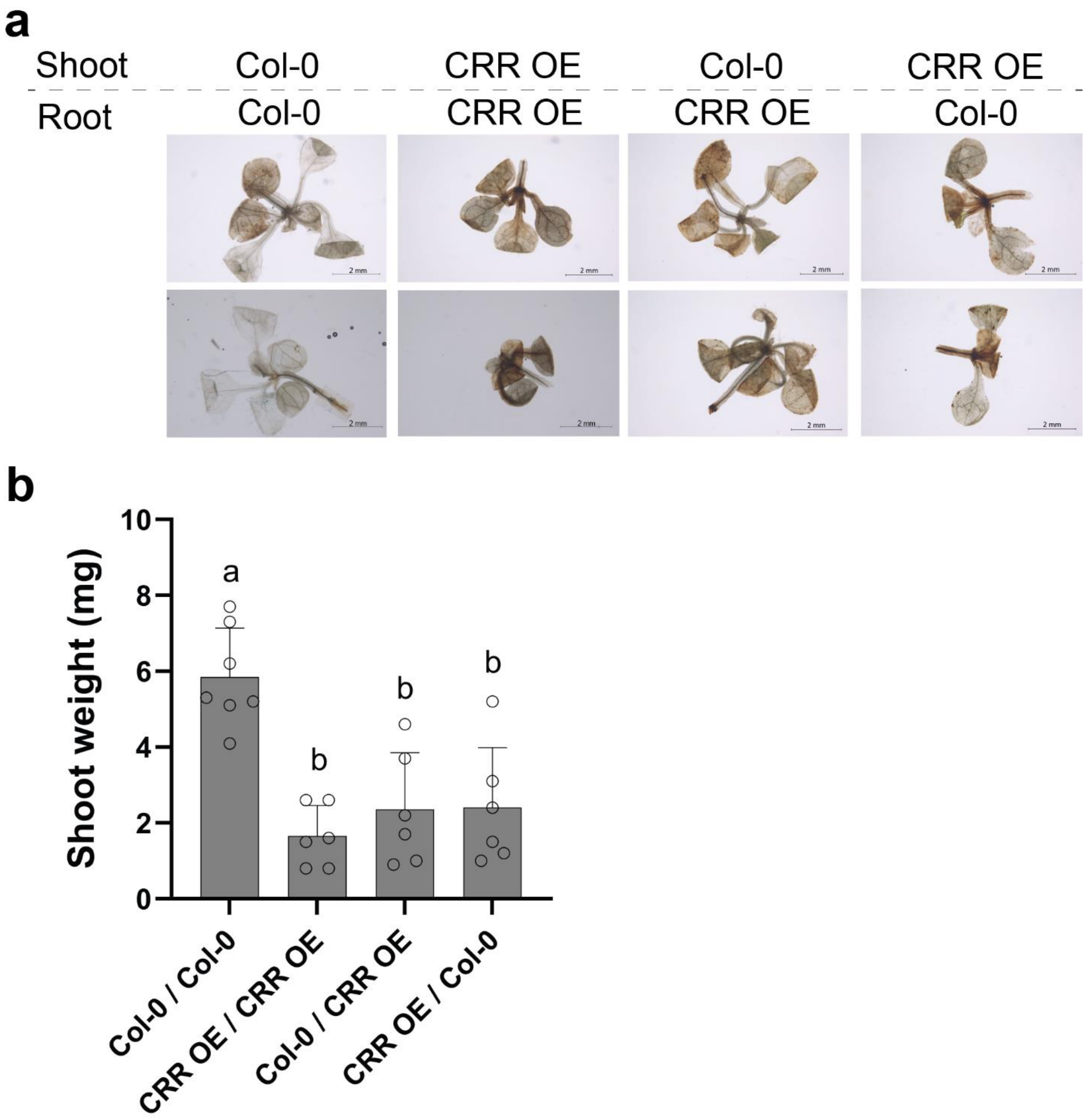

Supplementary Fig. 33 CRR over-expression in roots or shoots is sufficient to trigger iron hypersensitivity.

a, Homo- and hetero-grafts between Col-0 and CRR OE seedlings. Grafts were grown for 5 days on regeneration media and then transferred for 4 days to media containing high levels of iron (600 µM FeEDTA) and a Perls-DAB staining was performed. **b**, Shoot weight of the seedlings shown in (a). Data is represented as mean ± S.D. Different letters indicate significant differences between samples according to a one-way ANOVA followed by a Tukey's test, $p \leq 0.05$. Source data, including n and p values, are provided as a Source Data file.
