# Supplementary material for: A CYBDOM protein impacts iron homeostasis and primary root growth under phosphate deficiency in Arabidopsis
Source: Nat Commun. 2024 Jan 11;15:423. doi: 10.1038/s41467-023-43911-x (PMC10784552; doi:10.1038/s41467-023-43911-x)
Supplement: Supplementary file 3 — Description of Additional Supplementary Files [file 41467_2023_43911_MOESM3_ESM.pdf]

Description of Additional Supplementary Files

Supplementary Data 1 | Proteomic data obtained from Col-0 (WT) and *cnx1cnx2* double mutant grown in plus (+Pi) and minus (-Pi) phosphate.

Supplementary Data 2 | Reads number per sample obtained for *crr* mutant and Col0 after RNA sequencing. For each sample is shown the number of reads obtained, the number of reads after filtration and the percentage that was mapped to Arabidopsis genome.

Supplementary Data 3 | Reads number per sample obtained for CRR OE and Col-0 after RNA sequencing. For each sample is shown the number of reads obtained, the number of reads after filtration and the percentage that was mapped to Arabidopsis genome.

Supplementary Data 4 | Normalized expression values of Arabidopsis Col-0 and *crr* genes obtained by RNA-seq. For each Arabidopsis gene it is indicated the gene identification number (Gene_ID) and the normalized expression value in each condition expressed as CPM (counts per million reads) log2 transformed.

Supplementary Data 5 | Differential expression testing of Arabidopsis Col-0 and *crr* genes obtained by RNA-seq. For each pairwise comparison it is shown the gene Id, the mean expression value in the indicated condition, the log2 of the fold change (logFC), the p-value and the adjusted p-value (adj.P.Val). A moderated t-test was used for each contrast and an F-test was used for the interaction. For each group of comparisons, the adjusted p-value is computed by the Benjamini–Hochberg method, controlling for the false discovery rate (FDR or adj.P.Val).

Supplementary Data 6 | Normalized expression values of Arabidopsis Col-0 and CRR OE genes obtained by RNA-seq. For each Arabidopsis gene it is indicated the gene identification number (Gene_ID) and the normalized expression value in each condition expressed as CPM (counts per million reads) log2 transformed.

Supplementary Data 7 | Differential expression testing of Arabidopsis Col-0 and CRR OE genes obtained by RNA-seq. For each pairwise comparison it is shown the gene Id, the mean expression value in the indicated condition, the log2 of the fold change (logFC), the p-value and the adjusted p-value (adj.P.Val). A moderated t-test was used for each contrast and an F-test was used for the interaction. For each group of comparisons, the adjusted p-value is computed by the Benjamini–Hochberg method, controlling for the false discovery rate (FDR or adj.P.Val).

Supplementary Data 8 | Primer list. Sequence and description of the primers used in this work.
