# Supplementary material for: A CYBDOM protein impacts iron homeostasis and primary root growth under phosphate deficiency in Arabidopsis
Source: Nat Commun. 2024 Jan 11;15:423. doi: 10.1038/s41467-023-43911-x (PMC10784552; doi:10.1038/s41467-023-43911-x)
Supplement: Supplementary file 12 — Reporting Summary [file 41467_2023_43911_MOESM12_ESM.pdf]

nature portfolio

Corresponding author(s): Yves Poirier

Last updated by author(s): Nov 13, 2023

Reporting Summary

Nature Portfolio wishes to improve the reproducibility of the work that we publish. This form provides structure for consistency and transparency in reporting. For further information on Nature Portfolio policies, see our Editorial Policies and the Editorial Policy Checklist.

Statistics

For all statistical analyses, confirm that the following items are present in the figure legend, table legend, main text, or Methods section.

n/a	Confirmed	
☐	☒	The exact sample size (*n*) for each experimental group/condition, given as a discrete number and unit of measurement
☐	☒	A statement on whether measurements were taken from distinct samples or whether the same sample was measured repeatedly
☐	☒	The statistical test(s) used AND whether they are one- or two-sided<br>*Only common tests should be described solely by name; describe more complex techniques in the Methods section.*
☒	☐	A description of all covariates tested
☐	☒	A description of any assumptions or corrections, such as tests of normality and adjustment for multiple comparisons
☐	☒	A full description of the statistical parameters including central tendency (e.g. means) or other basic estimates (e.g. regression coefficient) AND variation (e.g. standard deviation) or associated estimates of uncertainty (e.g. confidence intervals)
☐	☒	For null hypothesis testing, the test statistic (e.g. F, t, r) with confidence intervals, effect sizes, degrees of freedom and P value noted<br>*Give P values as exact values whenever suitable.*
☒	☐	For Bayesian analysis, information on the choice of priors and Markov chain Monte Carlo settings
☒	☐	For hierarchical and complex designs, identification of the appropriate level for tests and full reporting of outcomes
☒	☐	Estimates of effect sizes (e.g. Cohen's *d*, Pearson's *r*), indicating how they were calculated

Our web collection on statistics for biologists contains articles on many of the points above.

Software and code

Policy information about availability of computer code

Data collection	No software was used for data collection
Data analysis	RNA seq analysis used the following publically available sofwares: Cutadapt v. 1.8 (Martin, 2011), fastq_screen v. 0.11.1 (https://www.bioinformatics.babraham.ac.uk/projects/fastq_screen/), reaper v. 15-065 (Davis et al., 2013), STAR v. 2.5.3a (Dobin et al., 2013), htseq-count v. 0.9.1 (Anders et al., 2015), RSeQC v. 2.3.7 (Wang et al., 2012), the EdgeR package version 3.24.3 (Robinson et al., 2010).<br>The analysis of confocal and bright field microscopy images was done with the ZEN 2 (blue edition) software from Zeiss and Fiji 2.9.0 (https:// imagej.net/software/fiji/).<br>Statistical analysis and graphs were generated with GraphPad Prism 9.<br>Structural superimposition and structure-based sequence alignment were performed with Chimera v.1.14 122. Electrostatic surface potentials were calculated with the PyMOL Molecular Graphics System, Version 2.0 Schrödinger, LLC<br>Secondary structure elements representation and residue conservation were generated with ESPript server (https://espript.ibcp.fr)<br>sgRNAs were designed using CRISPR-P V2 (http://crispr.hzau.edu.cn/CRISPR2/)

For manuscripts utilizing custom algorithms or software that are central to the research but not yet described in published literature, software must be made available to editors and reviewers. We strongly encourage code deposition in a community repository (e.g. GitHub). See the Nature Portfolio guidelines for submitting code & software for further information.

Data

Policy information about availability of data

All manuscripts must include a data availability statement. This statement should provide the following information, where applicable:

- Accession codes, unique identifiers, or web links for publicly available datasets
- A description of any restrictions on data availability
- For clinical datasets or third party data, please ensure that the statement adheres to our policy

Data associated with all figures and tables is provided in the Source Data file as well as Supplementary Data files. The RNA seq datasets for crr-1 and CRR OE have been deposited at the Gene Expression Omnibus (https://www.ncbi.nlm.nih.gov/geo/) (GSE235511 and GSE215755), while the proteomic data from this work has been deposited at ProteomeXchange Consortium via the PRIDE partner repository (http://www.ebi.ac.uk/pride) with the dataset identifier PXD040981. Plant lines will be made available upon request to the corresponding author.

Research involving human participants, their data, or biological material

Policy information about studies with human participants or human data. See also policy information about sex, gender (identity/presentation), and sexual orientation and race, ethnicity and racism.

Reporting on sex and gender	Not applicable
Reporting on race, ethnicity, or other socially relevant groupings	Not applicable
Population characteristics	Not applicable
Recruitment	Not applicable
Ethics oversight	Not applicable

Note that full information on the approval of the study protocol must also be provided in the manuscript.

Field-specific reporting

Please select the one below that is the best fit for your research. If you are not sure, read the appropriate sections before making your selection.

☒ Life sciences ☐ Behavioural & social sciences ☐ Ecological, evolutionary & environmental sciences

For a reference copy of the document with all sections, see nature.com/documents/nr-reporting-summary-flat.pdf

Life sciences study design

All studies must disclose on these points even when the disclosure is negative.

Sample size	No sample-size calculation was performed
Data exclusions	No data was excluded from analysis
Replication	All controls and samples in a single experiment were handled in parallel at the same time and conditions. In general, three or more biological replicates were performed in all experiments
Randomization	Samples from wild-type, mutants and transgenic plants were randomly collected for analysis. Petri dishes or pots where plants were grown, were randomly allocated in the growth chambers.
Blinding	Not relevant to this work

Reporting for specific materials, systems and methods

We require information from authors about some types of materials, experimental systems and methods used in many studies. Here, indicate whether each material, system or method listed is relevant to your study. If you are not sure if a list item applies to your research, read the appropriate section before selecting a response.

Materials & experimental systems

n/a	Involved in the study
☒	☐ Antibodies
☒	☐ Eukaryotic cell lines
☒	☐ Palaeontology and archaeology
☒	☐ Animals and other organisms
☒	☐ Clinical data
☒	☐ Dual use research of concern
☐	☒ Plants

Methods

n/a	Involved in the study
☒	☐ ChIP-seq
☒	☐ Flow cytometry
☒	☐ MRI-based neuroimaging

Dual use research of concern

Policy information about dual use research of concern

Hazards

Could the accidental, deliberate or reckless misuse of agents or technologies generated in the work, or the application of information presented in the manuscript, pose a threat to:

No	Yes
☒	☐ Public health
☒	☐ National security
☒	☐ Crops and/or livestock
☒	☐ Ecosystems
☒	☐ Any other significant area

Experiments of concern

Does the work involve any of these experiments of concern:

No	Yes
☒	☐ Demonstrate how to render a vaccine ineffective
☒	☐ Confer resistance to therapeutically useful antibiotics or antiviral agents
☒	☐ Enhance the virulence of a pathogen or render a nonpathogen virulent
☒	☐ Increase transmissibility of a pathogen
☒	☐ Alter the host range of a pathogen
☒	☐ Enable evasion of diagnostic/detection modalities
☒	☐ Enable the weaponization of a biological agent or toxin
☒	☐ Any other potentially harmful combination of experiments and agents

Plants

Seed stocks	The hyp1 mutant (At5g35735) was provided by Ricardo Fabiano Giehl (IPK, Gatersleben, Germany). The T-DNA insertion lines crr-1 (Salk_202530), crr-2 (Salkseq_039879.2), crr;h1 (Salk_016284, Salk_013527c), almt1 (Salk_009629) and lpr1 (Salk_016297) were obtained from the European Arabidopsis Stock Center (Nottingham, UK)
Novel plant genotypes	Novel plant genotypes were generated by Agrobacterium-mediated transformation and several independent lines were analyzed. All data reported was generated using T3 lines, with the exception of crr-1 complemented with cell type specific promoters, where T2 transgenic seeds selected by red fluorescence were used. CRR edited lines were generated using the CRISPR/CAS9 technology.
Authentication	T-DNA mutants were validated by PCR as described in the material and methods section section. CRISPR generated lines were analyzed by PCR amplification followed by sequencing. For CRR T-DNA mutants, transgenic lines generated in this study, and CRR-edited lines, at least two independent lines were analyzed
